# Immunogenicity and safety of fractional doses of yellow fever vaccines: a randomised, double-blind, non-inferiority trial

**DOI:** 10.1016/S0140-6736(20)32520-4

**Published:** 2021-01-09

**Authors:** Aitana Juan-Giner, Derick Kimathi, Kyra H Grantz, Mainga Hamaluba, Patrick Kazooba, Patricia Njuguna, Gamou Fall, Moussa Dia, Ndeye S Bob, Thomas P Monath, Alan D Barrett, Joachim Hombach, Edgar M Mulogo, Immaculate Ampeire, Henry K Karanja, Dan Nyehangane, Juliet Mwanga-Amumpaire, Derek A T Cummings, Philip Bejon, George M Warimwe, Rebecca F Grais

**Affiliations:** a Epicentre, Paris, France; b Kenya Medical Research Institute—Wellcome Trust Research Programme, Kilifi, Kenya; c Centre for Tropical Medicine & Global Health, University of Oxford, Oxford, UK; d Department of Biology, University of Florida, Gainesville, FL, USA; e Emerging Pathogens Institute, University of Florida, Gainesville, FL, USA; f Department of Epidemiology, Johns Hopkins Bloomberg School of Public Health, Baltimore, MD, USA; g Epicentre Mbarara Research Centre, Mbarara, Uganda; h Institut Pasteur Dakar, Dakar, Senegal; i Crozet BioPharma LLC, Devens, MA, USA; j Sealy Institute for Vaccines Sciences and Department of Pathology, University of Texas Medical Branch, Galveston, TX, USA; k Immunization, Vaccines & Biologicals, WHO, Geneva, Switzerland; l Department of Community Health, Mbarara University of Science & Technology, Mbarara, Uganda; m Expanded Program on Immunization, Ministry of Health, Kampala, Uganda

## Abstract

**Background:**

Stocks of yellow fever vaccine are insufficient to cover exceptional demands for outbreak response. Fractional dosing has shown efficacy, but evidence is limited to the 17DD substrain vaccine. We assessed the immunogenicity and safety of one-fifth fractional dose compared with standard dose of four WHO-prequalified yellow fever vaccines produced from three substrains.

**Methods:**

We did this randomised, double-blind, non-inferiority trial at research centres in Mbarara, Uganda, and Kilifi, Kenya. Eligible participants were aged 18–59 years, had no contraindications for vaccination, were not pregnant or lactating, had no history of yellow fever vaccination or infection, and did not require yellow fever vaccination for travel. Eligible participants were recruited from communities and randomly assigned to one of eight groups, corresponding to the four vaccines at standard or fractional dose. The vaccine was administered subcutaneously by nurses who were not masked to treatment, but participants and other study personnel were masked to vaccine allocation. The primary outcome was proportion of participants with seroconversion 28 days after vaccination. Seroconversion was defined as post-vaccination neutralising antibody titres at least 4 times pre-vaccination measurement measured by 50% plaque reduction neutralisation test (PRNT_50_). We defined non-inferiority as less than 10% decrease in seroconversion in fractional compared with standard dose groups 28 days after vaccination. The primary outcome was measured in the per-protocol population, and safety analyses included all vaccinated participants. This trial is registered with ClinicalTrials.gov, NCT02991495.

**Findings:**

Between Nov 6, 2017, and Feb 21, 2018, 1029 participants were assessed for inclusion. 69 people were ineligible, and 960 participants were enrolled and randomly assigned to vaccine manufacturer and dose (120 to Bio-Manguinhos-Fiocruz standard dose, 120 to Bio-Manguinhos-Fiocruz fractional dose, 120 to Chumakov Institute of Poliomyelitis and Viral Encephalitides standard dose, 120 to Chumakov Institute of Poliomyelitis and Viral Encephalitides fractional dose, 120 to Institut Pasteur Dakar standard dose, 120 to Institut Pasteur Dakar fractional dose, 120 to Sanofi Pasteur standard dose, and 120 to Sanofi Pasteur fractional dose). 49 participants had detectable PRNT_50_ at baseline and 11 had missing PRNT_50_ results at baseline or 28 days. 900 were included in the per-protocol analysis. 959 participants were included in the safety analysis. The absolute difference in seroconversion between fractional and standard doses by vaccine was 1·71% (95% CI −2·60 to 5·28) for Bio-Manguinhos-Fiocruz, −0·90% (–4·24 to 3·13) for Chumakov Institute of Poliomyelitis and Viral Encephalitides, 1·82% (–2·75 to 5·39) for Institut Pasteur Dakar, and 0·0% (–3·32 to 3·29) for Sanofi Pasteur. Fractional doses from all four vaccines met the non-inferiority criterion. The most common treatment-related adverse events were headache (22·2%), fatigue (13·7%), myalgia (13·3%) and self-reported fever (9·0%). There were no study-vaccine related serious adverse events.

**Interpretation:**

Fractional doses of all WHO-prequalified yellow fever vaccines were non-inferior to the standard dose in inducing seroconversion 28 days after vaccination, with no major safety concerns. These results support the use of fractional dosage in the general adult population for outbreak response in situations of vaccine shortage.

**Funding:**

The study was funded by Médecins Sans Frontières Foundation, Wellcome Trust (grant no. 092654), and the UK Department for International Development. Vaccines were donated in kind.

## Introduction

Yellow fever is a mosquito-borne viral disease that is endemic in 44 countries.^1^ Four live attenuated yellow fever virus vaccines derived from the 17D strain are WHO-prequalified, including 17DD from Bio-Manguinhos-Fiocruz (Brazil), 17D-213 from Federal State Unitary Enterprise of Chumakov Institute of Poliomyelitis and Viral Encephalitides (Russia), and 17D-204 from Institut Pasteur Dakar (Senegal) and Sanofi Pasteur (France). All four vaccines have been widely used and are considered safe and effective.^1^ WHO recommends routine vaccination in all countries in which yellow fever is endemic, vaccination of travellers to those areas, and mass vaccination for prevention or control of outbreaks. A stockpile of 2 million doses was reserved for outbreak response in 2000, and was increased to 6 million in 2014.^2^

**Research in context**

**Evidence before this study**

In July, 2016, after major yellow fever outbreaks in Angola and DR Congo, WHO published a secretariat information paper including a review of studies assessing the immunogenicity of fractional doses of yellow fever vaccines, and recommended consideration of fractional doses to manage a vaccine shortage. Fractional doses of yellow fever vaccine produced by Bio-Manguinhos-Fiocruz (17DD substrain) were given to approximately 7·5 million non-pregnant adults and children aged 2 years or older in Kinshasa, DR Congo. The evidence to support this action was limited to a single vaccine substrain and to a specific context. To broaden and simplify recommendations, WHO called for additional research to be done.

**Added value of this study**

This is the first randomised controlled trial assessing all four WHO-prequalified yellow fever vaccines, providing information on the immunogenicity and safety of fractional doses of the vaccine substrains at 10 days, 28 days, and 1 year post-vaccination. The results show that, at 28 days post-vaccination, most participants had high neutralising antibodies and that seroconversion rates in the fractional dose groups were non-inferior to standard dose for all vaccines. Seroconversion rates and neutralising antibodies remained high up to 1 year post-vaccination for both fractional and standard doses for all vaccines. These results are aligned with previous studies using the 17DD substrain vaccine but extend the evidence to randomised comparisons of all four vaccines and to sub-Saharan Africa.

**Implications of all the available evidence**

Our study supports the use of one-fifth fractional doses of all four WHO-prequalified yellow fever vaccines for the general adult population and fills a crucial knowledge gap to support WHO policy on the use of fractional dosing of yellow fever vaccine for outbreak response. The immunogenicity and safety of fractional dosing in children and specific populations, such as those living with HIV, is yet to be established. Long-term studies are warranted to substantiate the duration of protection.

An outbreak of yellow fever in 2016 in Angola raised major concerns about the adequacy of vaccine supply. Routine vaccination was suspended in some African countries to meet demand,^3^ and a subsequent outbreak in DR Congo added to the global shortage.^4^ In response, WHO reviewed the evidence on fractional dosing of yellow fever vaccine as a dose-sparing option and recommended exceptional consideration of off-label use as fractional doses, administered by the standard subcutaneous or intramuscular route, to extend supply.^4^, ^5^ A fractional dose constituting a fifth of the standard dose of the yellow fever vaccine produced by Bio-Manguinhos-Fiocruz (17DD substrain) was given to approximately 7·5 million non-pregnant adults and children aged 2 years or older in Kinshasa, DR Congo in August, 2016.^6^ Fractional dosing was again used in response to outbreaks in 2017–18 in the states of São Paulo, Rio de Janeiro, and Bahia, Brazil, and given to almost 17 million people.^7^

Four studies^8^, ^9^, ^10^, ^11^ have assessed the immunogenicity of fractional doses of yellow fever vaccine. One^8^ used an old vaccine formulation that is no longer produced, and another focused on intradermal administration.^9^ The evidence to support WHO recommendations was primarily from a dose-finding study^10^ of the 17DD substrain vaccine in healthy male army recruits in Brazil that showed seroconversion rates greater than 97% with doses as low as 587 IU/dose, and similar virological and immunological kinetics with doses down to 3013 IU/dose, compared with the standard dose of 27 476 IU/dose.^11^ Historical data^12^ suggest that standard doses of yellow fever vaccines should contain at least 1000 IU/dose. However, release specifications vary by manufacturer, are related to the stability of the vaccine, and are generally several times higher than the minimum specification.^13^ WHO recommend that the minimum dose administered, standard or fractional, should contain 3000 IU/dose, and the decision whether to use one half or one fifth of the standard dose should consider the potency of the vaccine batch.^4^ During the fractional dose campaign in Kinshasa, DR Congo, a study^14^ supported the immunogenicity of fractional doses of the 17DD substrain vaccine in a large-scale campaign. The dose-finding study in Brazil^10^, ^11^ also included only 17DD, and no information is available for the remaining WHO-prequalified vaccines.

We assessed, for the first time, the immunogenicity and safety of fractional doses of all four WHO-prequalified yellow fever vaccines, to provide evidence to broaden recommendations to include all WHO-prequalified yellow fever vaccines for fractional dosing.^4^

## Methods

### Study design

We did this double-blind, individually-randomised non-inferiority trial at the Epicentre Mbarara Research Centre in Mbarara, Uganda, and the Kenya Medical Research Institute-Wellcome Trust Research Programme clinical trials facility in Kilifi, Kenya.^15^ Mbarara district is located in proximity to Masaka, Rukungiri, and Kalangala districts, all of which registered confirmed yellow fever cases in 2016.^16^

The study protocol^15^ was approved by Research Ethics Review Committee of WHO (Switzerland); Scientific & Ethics Review Unit, Kenya Medical Research Institute (Kenya); Oxford Tropical Research Ethics Committee (United Kingdom); Mbarara University of Science and Technology Research Ethics Committee (Uganda); and the Uganda National Council of Sciences and Technology (Uganda). Approval was obtained from the national regulatory authorities in Uganda and Kenya. The trial was done in accordance with Good Clinical Practice guidelines.

### Participants

Participants were recruited from rural communities in the Mbarara municipality, Mbarara district, Uganda, and Kilifi County, Kenya. Communities were informed about the trial using locally adapted strategies. Individuals interested in participating were invited to the study sites. Written informed consent was required to participate. Eligible participants were aged 18–59 years, had no contraindications for vaccination, were not pregnant or lactating, had no history of previous yellow fever vaccination or infection, did not require yellow fever vaccination for travel, and were able to comply with study procedures.

### Randomisation and masking

Participants were randomly assigned to one of eight equal groups corresponding to the four prequalified yellow fever vaccines at standard or fractional dose. Unique allocation numbers were prepared by an independent statistician (DiagnoSearch LifeSciences, Mumbai, India) using a computer-generated random number list with non-disclosed fixed blocks of size 10, with equal allocation to dose within a block and to a given manufacturer by site. The allocation sequence was concealed using pre-prepared, sequentially numbered scratch-off booklets that were stored securely. After enrolment, the vaccination nurse scratched off the randomisation code indicating the allocated vaccine and dose for the participant. Vaccines were reconstituted and administered in a private room not accessible to other study staff.

Participants were masked by covering the volume of the syringe with opaque tape. The vaccination nurse and supervisor overseeing vaccination were aware of allocation; personnel assessing outcomes and investigators were masked to vaccine and dose throughout.

### Procedures

A batch of standard 10-dose vials of yellow fever vaccine was selected from each manufacturer with potency at time of release closest to internal minimum specification. Vaccine potency was independently measured at the National Institute for Biological Standards and Control (UK; table 1). The freeze-dried preparations and diluents were kept at 2–8°C until administration. After each participant was randomly assigned, a vaccine vial was reconstituted using the manufacturer's diluent. A syringe was prepared immediately before vaccination. Reconstituted vaccines were kept in a vaccine carrier at 2–8°C as per WHO and manufacturer requirements. Any remaining reconstituted vaccine was discarded after 6 h. Fractional doses consisted of one fifth (0·1 ml) of the standard dose (0·5 ml). Vaccine was administered subcutaneously in the deltoid region using 0·5 ml auto-disable syringes (needle size 25G × 3/4”) with a 45° injection angle for the standard 0·5 ml dose and 0·1 ml auto-disable syringes (needle size 26G × 3/8”) at 90° injection angle for the 0·1 ml fractional dose.

**Table 1:** Characteristics of study vaccines by manufacturer

	**Substrain**	**Expiry date**	**IU/dose mean (SD)**
Bio-Manguinhos-Fiocruz	17DD	Oct 31, 2018	38 905 (1·26)
Chumakov Institute of Poliomyelitis and Viral Encephalitides	17D-213	Feb 28, 2018	43 652 (1·12)
Institut Pasteur Dakar	17D-204	March 31, 2019	6761 (1·74)
Sanofi Pasteur	17D-204	Feb 28, 2019	16 596 (1·26)

Standard doses of the vaccines were tested at the National Institute for Biological Standards and Control, UK, in December, 2017. The IU/dose is the mean of three assays.

Participants were followed up at 10 days (±1 day), 28 days (±3 days), and 365 days (±14 days) after vaccination. Participants had a medical consultation at each study visit, and a blood sample was obtained at the first visit (before vaccination) and at each scheduled follow-up visit.

Serum samples were separated and aliquoted into 3 samples within 4 h after obtention and stored in −80°C freezers at the study sites. Serum samples were analysed at the Institut Pasteur Dakar (Senegal), where neutralising antibody titres against yellow fever were assessed by 50% and 90% plaque reduction neutralisation tests (PRNT_50_ and PRNT_90_).^17^, ^18^ Laboratory personnel were masked to vaccine and dose allocation.

The attenuated yellow fever vaccine 17D-204 from Institut Pasteur Dakar was used as the challenge strain for the neutralisation assays. Viral stock preparation was in C6/36 cells and titrated by plaque assay method previously described.^19^ A defined virus concentration of 10^3^ plaque forming units per ml, and diluted and heat-inactivated serum samples were titrated in serial two-fold dilutions from 1:10 to 1:20480. Serum and virus were mixed and incubated before overlaying with 0·6% carboxymethylcellulose and 3% fetal calf serum in L-15 Leibovitz's media. After 4–5 days of incubation at 37°C, plaques were counted, and antibody titre was defined as the dilution that reduced observed plaques by 50% and 90% compared with that observed in control wells.

### Outcomes

The primary outcome was non-inferiority in the proportion of participants with seroconversion, as determined by PRNT_50_, by day 28 after vaccination for fractional dose compared with standard dose for each vaccine. Seroconversion was defined as a post-vaccination rise in neutralising antibody titre of at least four times the pre-vaccination sample.

Secondary outcomes at day 28 after vaccination included assessment of geometric mean titres (GMT) and geometric mean fold increase (GMFI; ie, geometric mean of the ratios of post-vaccination titre to pre-vaccination titre). Seroconversion, GMT, and GMFI were also assessed at 10 days and 1 year after vaccination to assess the rapidity of protection and the lasting effect of vaccination. Safety outcomes were also included as secondary outcomes, and included assessment of adverse events (AEs) for 28 days after vaccination, and serious adverse events (SAEs) throughout follow-up. SAEs were defined as any new health-related problem that occurred during follow-up and resulted in death, was life-threatening, necessitated hospital admission or prolongation of existing hospital stay, or resulted in disability or incapacity. AEs included all untoward medical events and were evaluated in the 30 minutes following vaccination and up to 28 days after vaccination. At the visits at 10 and 28 days after vaccination, a clinician asked for the presence of local reaction, headache, fatigue, muscle pain, fever, gastrointestinal problem, and any other symptom since the previous visit. Participants were also asked to report any other symptoms or concerns during and outside scheduled visits. We recorded the nature, relatedness, severity, and outcome of each AE.^20^, ^21^ All events were coded using Medical Dictionary for Regulatory Activities (MedDRA), v20.0.

### Statistical analysis

To weigh the public health consequence of loss of protection against an increase in available vaccine doses and coverage, non-inferiority was defined as seroconversion 28 days after vaccination no more than 10% less with the fractional dose than with the standard dose. We assumed 95% seroconversion with standard doses and considered that a loss of protection to 85% seroconversion with fractional doses would still ensure protection above 80%, which is necessary to interrupt local transmission.^22^ The non-inferiority margin was supported by a modelling study of yellow fever vaccination,^23^ in which fractional doses with 80–90% efficacy were beneficial in high-transmission areas. To detect a non-inferiority margin of 10% in seroconversion 28 days after vaccination, with a 2·5% significance level for a one-sided test, 90% power, and accounting for 5% loss to follow-up and 15% baseline yellow fever seropositivity (ie, PRNT_50_ titre ≥10), a sample size of 120 people per unique dose was required (240 per manufacturer). 960 participants were to be recruited (480 per study site).

Analysis consisted of pairwise comparisons of fractional versus standard dose of the same manufacturer's vaccine. No comparisons were made between vaccines from different manufacturers. Comparison of baseline characteristics were done using χ^2^ tests (or Fisher's exact test for smallest count <5) for categorical variables and Student's t-test for continuous variables. Any PRNT_50_ titre reported as seronegative (limit of quantification [LOQ] <10) was converted to LOQ/2. Any PRNT_50_ titre greater than 20 480 was designated 20 480, as this was the limit of quantification of titres or the last serial dilution.

The number and percentage of participants who seroconverted are presented by manufacturer and dose with two-sided exact Clopper-Pearson 95% CI. Non-inferiority for the primary outcome was assessed by constructing a two-sided 95% CI using the Wilson score interval of the point difference between seroconversion rates in the fractional and standard dose arms. Fractional doses were considered non-inferior if the lower bound of the CI for difference in seroconversion was greater than −10%.

Two-sided 95% CIs of the mean difference between log GMT and log GMFI between the standard and fractional dose of each vaccine were generated using the *t*-distribution. Intervals were transformed to show the ratio of GMT and GMFI for the fractional dose compared with standard dose.

Immunogenicity outcomes were assessed in the per-protocol population and the intention-to-treat population. The per-protocol population included all participants for whom the eligibility criteria were appropriately applied, who were seronegative at baseline (PRNT_50_ <LOQ), and who had a PRNT_50_ titre at the time of interest. The intention-to-treat population included any vaccinated participant with at least one PRNT_50_ result after vaccination.

AEs and SAEs were summarised as number and percentage by study group. Safety outcomes were assessed in all vaccinated participants.

Data analysis was done in R, version 3.6.1. A data safety monitoring board regularly reviewed study data. This study is registered with ClinicalTrials.gov, NCT02991495.

### Role of the funding source

The funder of the study had no role in study design, data collection, data analysis, data interpretation, or writing of the report. The corresponding author had full access to all data in the study and had final responsibility for the decision to submit for publication.

## Results

Between Nov 6, 2017, and Feb 21, 2018, 1029 participants were assessed for eligibility. 69 people were ineligible (54 did not meet inclusion criteria, one declined to participate, three had underlying disease and 11 did not complete screening). 960 eligible participants were enrolled and randomly assigned to a vaccine manufacturer and dosage (120 to Bio-Manguinhos-Fiocruz standard dose, 120 to Manguinhos-Fiocruz fractional dose, 120 to Chumakov Institute of Poliomyelitis and Viral Encephalitides standard dose, 120 to Chumakov Institute of Poliomyelitis and Viral Encephalitides fractional dose, 120 to Institut Pasteur Dakar standard dose, 120 to Institut Pasteur Dakar fractional dose, 120 to Sanofi Pasteur standard dose, and 120 to Sanofi Pasteur fractional dose; figure). Participant characteristics are presented in table 2. The mean age of participants was 35·7 years at enrolment, and 55·1% of participants were female.

**Figure:**
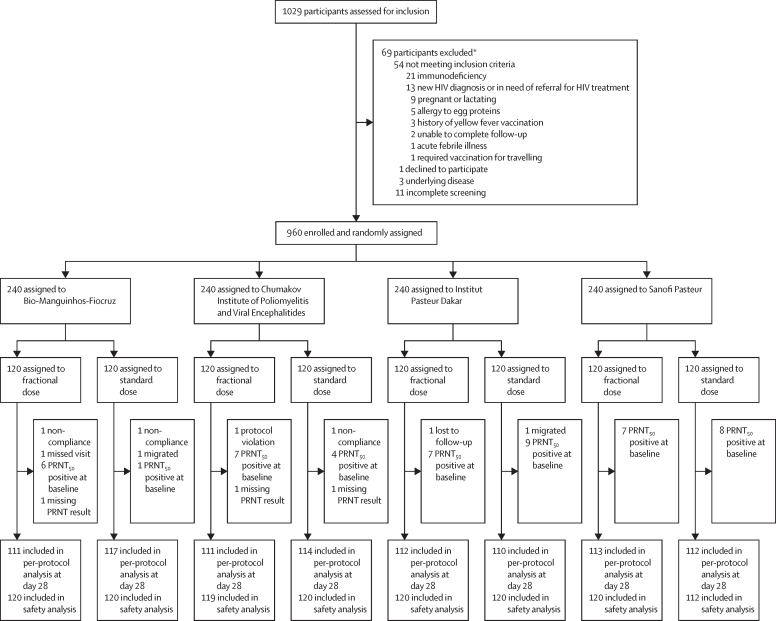
Trial profile PRNT_50_=50% plaque reduction neutralisation test. *Some participants were excluded according to more than one eligibility criterion.

**Table 2:** Baseline demographic and clinical characteristics

		**Bio-Manguinhos-Fiocruz**		**Chumakov Institute of Poliomyelitis and Viral Encephalitides**		**Institut Pasteur Dakar**		**Sanofi Pasteur**	
		Fractional (n=120)	Standard (n=120)	Fractional (n=119)	Standard (n=120)	Fractional (n=120)	Standard (n=120)	Fractional (n=120)	Standard (n=120)
Age, years		34·5 (11·5)	35·0 (11·1)	37·2 (11·4)	35·9 (11·8)	35·5 (11·4)	35·4 (11·7)	35·3 (11·7)	36·8 (12·9)
Sex									
	Female	73 (60·8%)	73 (60·8%)	58 (48·7%)	62 (51·7%)	64 (53·3%)	72 (60·0%)	61 (50·8%)	66 (55·0%)
	Male	47 (39·2%)	47 (39·2%)	61 (51·3%)	58 (48·3%)	56 (46·7%)	48 (40·0%)	59 (49·2%)	54 (45·0%)
HIV positive		4 (3·3%)	1 (0·8%)	0	0	1 (0·8%)	1 (0·8%)	1 (0·8%)	2 (1·7%)
Seropositive to yellow fever		6* (5·0%)	1 (0·8%)	7* (5·9%)	4† (3·4%)	7 (5·8%)	9 (7·5%)	7 (5·8%)	8 (6·7%)

Data are mean (SD) or n (%). PRNT_50_=50% plaque reduction neutralisation test.

* Total n=118 due to missing PRNT titre at baseline.

† Total n=119 due to missing PRNT titre at baseline. Seropositive to yellow fever at baseline defined as PRNT_50_ titre ≥10.

952 (99·2%) of 960 participants completed the 28 days post-vaccination visit and 928 (96·7%) completed the 1 year post-vaccination visit. The most frequent reasons for discontinuation were migration out of study area (n=13), loss to follow-up (n=10) and non-compliance with study visits (n=4). Two participants discontinued due to protocol violation (one 65-year-old man, one re-vaccination of a participant attending the 28 days post-vaccination visit). The primary analysis (per protocol) 28 days post-vaccination included 900 (93·6%) of 960 randomly assigned participants. 49 participants had detectable PRNT_50_ at baseline (included in intention-to-treat analysis) and 11 participants had missing PRNT_50_ results at baseline or 28 days follow-up (figure).

28 days after vaccination, seroconversion rates were high in all groups, with at least 98·2% of participants seroconverting in every group (table 3). The difference in seroconversion between fractional and standard dose groups was 1·71% (95% CI −2·60 to 5·28) for the vaccine produced by Bio-Manguinhos-Fiocruz, −0·90% (–4·24 to 3·13) for the vaccine produced by Chumakov Institute of Poliomyelitis and Viral Encephalitides, 1·82% (–2·75 to 5·39) for the vaccine produced by Institut Pasteur Dakar, and 0·0% (–3·32 to 3·29) for the vaccine produced by Sanofi Pasteur. The lower bound of the 95% CI for the difference in seroconversion between fractional and standard dose groups for each vaccine excluded the defined non-inferiority margin of −10%, indicating non-inferiority of the fractional dose (table 3). Non-inferiority was also met with PRNT_90_ results (appendix p 6). Results for the intention-to-treat population at 28 days after vaccination were similar, with a lower bound of the CI for the difference in seroconversion between fractional and standard dose groups for each vaccine of −2·58% for the vaccine produced by Bio-Manguinhos-Fiocruz, −6·11% for the vaccine produced by Chumakov Institute of Poliomyelitis and Viral Encephalitides, −2·11% for the vaccine produced by Institut Pasteur Dakar, and −3·10% for the vaccine produced by Sanofi Pasteur (appendix p 7). Seroconversion was high even among adults with neutralising antibodies against yellow fever at baseline (appendix p 7).

**Table 3:** Seroconversion and geometric mean titre by PRNT_50_ in fractional and standard dose of yellow fever vaccine at day 10, day 28, and day 365 post-vaccination in the per-protocol population, by vaccine manufacturer

		**Total (n)**	**Seroconversion**			**Geometric mean titre**	
			**n**	**% (95% CI)**	**Difference (fractional–standard)**	**Titre (95% CI)**	**Ratio (fractional:standard)**
**Bio-Manguinhos-Fiocruz**							
Day 10							
	Fractional	110	68	61·8 (52·1 to 70·9)	..	51·1 (35·4 to 74·0)	..
	Standard	117	69	59·0 (49·5 to 68·0)	2·84 (−9·60 to 15·5)	43·2 (30·2 to 61·7)	1·18 (0·71 to 1·97)
Day 28							
	Fractional	111	111	100·0 (96·7 to 100·0)	..	3939 (2812, 5516)	..
	Standard	117	115	98·3 (94·0 to 99·8)	1·71 (−2·60 to 5·28)	4064 (2850 to 5794)	0·97 (0·60 to 1·58)
Day 365							
	Fractional	110	110	100·0 (96·7 to 100·0)	..	3508 (2492 to 4939)	..
	Standard	111	110	99·1 (95·1 to 100·0)	0·90 (−3·13 to 4·36)	2708 (1877 to 3908)	1·30 (0·79 to 2·13)
**Chumakov Institute of Poliomyelitis and Viral Encephalitides**							
Day 10							
	Fractional	111	59	53·2 (43·4 to 62·7)	..	36·0 (24·6 to 52·6)	..
	Standard	114	70	61·4 (51·9 to 70·4)	−8·25 (−21·1 to 4·25)	54·5 (38·0 to 78·2)	0·66 (0·39 to 1·11)
Day 28							
	Fractional	111	110	99·1 (95·1 to 100·0)	..	5874 (4162 to 8289)	..
	Standard	114	114	100·0 (96·8 to 100·0)	−0·90 (−4·24 to 3·13)	5817 (4297 to 7876)	1·01 (0·64 to 1·59)
Day 365							
	Fractional	107	105	98·1 (93·4 to 100·0)	..	4081 (2848 to 5849)	..
	Standard	112	112	100·0 (96·8 to 100·0)	−1·87 (−5·45 to 2·82)	3757 (2672 to 5284)	1·09 (0·66 to 1·78)
**Institut Pasteur Dakar**							
Day 10							
	Fractional	112	70	62·5 (52·9 to 71·5)	..	48·5 (33·3 to 70·5)	..
	Standard	108	74	68·5 (58·9 to 77·1)	−6·02 (−18·5 to 6·20)	84·8 (55·4 to 129·7)	0·57 (0·33 to 1·00)
Day 28							
	Fractional	112	112	100·0 (96·8 to 100·0)	..	4279 (3182 to 5753)	..
	Standard	110	108	98·2 (93·6 to 99·8)	1·82 (−2·75 to 5·39)	2576 (1788 to 3712)	1·66 (1·04 to 2·65)
Day 365							
	Fractional	111	111	100·0 (96·7 to 100·0)	..	2974 (2148 to 4117)	..
	Standard	106	105	99·1 (94·9 to 100·0)	0·94 (−3·26 to 4·38)	2261 (1590 to 3214)	1·32 (0·82 to 2·12)
**Sanofi Pasteur**							
Day 10							
	Fractional	113	78	69·0 (59·6 to 77·4)	..	71·6 (49·1 to 104·4)	..
	Standard	111	86	77·5 (68·6 to 84·9)	−8·45 (−20·1 to 2·84)	104·0 (70·7 to 152·9)	0·69 (0·40 to 1·18)
Day 28							
	Fractional	113	113	100·0 (96·8 to 100·0)	..	5545 (4106, 7488)	..
	Standard	112	112	100·0 (96·8 to 100·0)	0·0 (−3·32 to 3·29)	4252 (3033 to 5961)	1·30 (0·83 to 2·04)
Day 365							
	Fractional	109	107	98·2 (93·5 to 99·8)	..	5088 (3724 to 6950)	..
	Standard	109	109	100·0 (96·7 to 100·0)	−1·83 (−5·49 to 2·77)	4047 (2989 to 5479)	−1·26 (−0·82 to 1·94)

PRNT_50_=50% plaque reduction neutralisation test. Data are n, % (95% CI), or n (95% CI).

10 days after vaccination, the percentage of participants with seroconversion varied from 53·2% to 77·5% between groups, with large and overlapping CIs (table 3). Seroconversion rates remained high 1 year after vaccination, with PRNT_50_ titre at least four times that of baseline in 98–100% of participants who received a fractional dose and more than 99% of those who received the standard dose (table 3).

GMTs of neutralising antibodies 28 days after vaccination were high in all groups, with titres ranging from 3939 to 5874 in the fractional dose groups and 2576 to 5817 in the standard dose groups. At 10 days after vaccination, GMTs in all groups were lower than at 28 days post-vaccination. Also at 10 days post-vaccination, GMTs were lower in the fractional dose groups than in the standard dose groups for all vaccines except for the vaccine manufactured by Bio-Manguinhos-Fiocruz, although the CIs for the ratio were wide and overlapped 1 for all vaccines (table 3). At 1 year post-vaccination, GMTs remained high in all study groups and were consistently higher in the fractional dose groups (range 2974–5088) compared with standard dose groups (2261–4047). There was some evidence for a slow decline in GMT from 28 days to 1 year post-vaccination in all groups (appendix p 14).

GMFIs showed an increase in neutralising antibody titres from baseline at each timepoint for all vaccine groups, with GMFIs reaching their highest point 28 days post-vaccination (appendix, pp 15–16).

AEs were common, with 48–63% of participants in each group reporting at least one AE and 32–49% reporting an AE classified as vaccine-related within 28 days of vaccination. There were similar number of events across all groups. The most common related AEs were headache (213 [22·2%] of 959), fatigue (131 [13·7%]), myalgia (128 [13·3%]), and pyrexia (86 [9·0%]; table 4, appendix pp 17–20). Ten SAEs were reported, including three deaths. All were classified as not related to the study vaccines (appendix p 20).

**Table 4:** Most common adverse events until day 28 post-vaccination by vaccine manufacturer in all vaccinated participants

	**Bio-Manguinhos-Fiocruz**		**Chumakov Institute of Poliomyelitis and Viral Encephalitides**		**Institut Pasteur Dakar**		**Sanofi Pasteur**	
	Fractional (n=120)	Standard (n=120)	Fractional (n=119)	Standard (n=120)	Fractional (n=120)	Standard (n=120)	Fractional (n=120)	Standard (n=120)
Headache	21 (17·5%)	26 (21·7%)	27 (22·7%)	30 (25·0%)	30 (25·0%)	22 (18·3%)	29 (24·2%)	28 (23·3%)
Fatigue	8 (6·7%)	15 (12·5%)	18 (15·1%)	12 (10·0%)	19 (15·8%)	21 (17·5%)	21 (17·5%)	17 (14·2%)
Myalgia	15 (12·5%)	18 (15·0%)	14 (11·8%)	15 (12·5%)	14 (11·7%)	22 (18·3%)	14 (11·7%)	16 (13·3%)
Pyrexia	9 (7·5%)	11 (9·2%)	12 (10·1%)	9 (7·5%)	12 (10·0%)	9 (7·5%)	10 (8·3%)	14 (11·7%)
Abdominal pain	4 (3·3%)	3 (2·5%)	3 (2·5%)	8 (6·7%)	9 (7·5%)	1 (0·8%)	6 (5·0%)	3 (2·5%)

Data are n (%). MedDRA=Medical Dictionary for Regulatory Activities. Events are MedDRA version 20.0 preferred term. A table with all reported adverse events up to day 28 post-vaccination by MedDRA coding, study group and vaccine manufacturer, is presented in the appendix (pp 17–20).

## Discussion

One-fifth fractional doses of the four WHO-prequalified yellow fever vaccines were non-inferior in seroconversion 28 days post-vaccination compared with the standard dose. Non-human studies^24^ suggest that neutralising titres of at least 40 protect against lethal yellow fever infection, and this has been used as the cutoff for protection from infection in humans. At 28 days post-vaccination, almost all participants showed neutralising antibodies far in excess of this assumed protective threshold.^24^ Seroconversion rates and GMT remained high up to 1 year after vaccination for both fractional and standard doses for all vaccines. There were no major safety concerns with either the standard or fractional dose of any of the four vaccines.

The results of our trial are aligned with previous studies on fractional dosing^10^, ^14^ using the 17DD substrain vaccine, indicating that nearly all vaccinated individuals seroconvert within 28 days of vaccination. We extend the evidence base to randomised comparisons for all WHO-prequalified vaccines and to a general adult population in rural sub-Saharan Africa. Previous studies^14^, ^25^, ^26^ indicate that the immune response generated by fractional doses is long-lived, which is consistent with our findings of a robust immunogenic response 1 year after vaccination, although longer-term studies are warranted.

For the first time in a randomised trial including all four WHO-prequalified yellow fever vaccines, we assessed seroconversion rates at 10 days post-vaccination in all participants. These results show overall lower seroconversion and GMTs in the fractional dose groups, suggesting a possible delayed immune response of the fractional doses. However, previous studies have shown that 80–90% of standard dose vaccine recipients have a protective concentration of neutralising antibodies 10 days after vaccination.^27^ This proportion was lower in our study, even in participants who received a standard dose of vaccine. Our study was done in African people, who might have lower B-cell and T-cell responses to yellow fever vaccination than European populations.^28^ Given the probable use of fractional doses in outbreak response, the low rates of seroconversion at 10 days are concerning and emphasise the need for early vaccination campaigns in outbreak response. Current International Health Regulations allow for travel 10 days after vaccination, and might require further study to fully understand implications for standard dose vaccination and any future recommendations for fractional doses.

The results of this study are subject to several limitations. First, the study was insufficiently powered to assess non-inferiority of the fractional doses at 10 days post-vaccination. 67% of participants seroconverted in the standard dose groups at 10 days after vaccination. Due to the low rates of seroconversion our study has only 35% power to detect a −10% non-inferiority margin difference between fractional and standard dose groups at 10 days post-vaccination, which is insufficient to draw conclusions on non-inferiority, and additional studies might be warranted to better understand the early immune response to vaccination with fractional doses. Second, we did not assess for presence of neutralising antibodies against other flaviviruses that could potentially interfere with the response to yellow fever vaccine. To compensate for this, participants were asked about history of infection with Zika, dengue or West Nile virus, but only one participant reported being aware of a previous infection. In total, only 49 participants had neutralising antibodies against yellow fever at baseline, which is very few. These participants were excluded from the per-protocol analysis. We did supplementary analyses at day 10, day 28, and day 365 including participants who were seropositive at baseline and found no difference in interpretation.

Finally, the primary limitation for the generalisability of results are the vaccines used in the study. We used vaccines as close as possible to each manufacturer's internal minimum specification for potency. Vaccines titres were high, however, with potencies 6–43 times the minimum specification established by WHO. All fractional doses still contained potencies above the minimum specification of 1000 IU. Vaccines also differed in expiry dates and had between 3 and 16 months of remaining shelf-life at the time of testing at the National Institute for Biological Standards and Control (table 1). The yellow fever vaccines in current use were developed more than 80 years ago^1^ following a production process that has not changed substantially since, and without clinical data to support minimum specifications.^13^ Historically, vaccines have been released at higher titres than the recommended minimum, partly to account for the loss of potency during shelf-life.^13^ Average doses of WHO-prequalified vaccines vary between 12 874 and 43 651 IU and consequently, titres of fractional doses would still exceed WHO minimum specification in principle.^29^ We consider that the batches selected for this study represent the WHO-prequalified vaccines, and that results should be generalisable to other batches if there is not a substantial change in the manufacturing process. Therefore, our study provides additional confidence in using fractional doses for the vaccines currently produced.

Given the longstanding practice of releasing batches with high potency, and the dependence on historical precedent for dose requirements, there was much uncertainty regarding the use of fractional dosing, even for doses above the minimum specifications. This is reflected in the WHO recommendations for fractional dosing that the minimal dose administered should preferentially contain 3000 IU/dose, three times the minimum specification for vaccine potency.^4^ Our data suggest that, even at the lower end of the range of vaccine potency and with little remaining shelf-life, fractional doses can be an option for outbreak response.

These results support the use of one-fifth fractional doses of the four WHO-prequalified yellow fever vaccines for the general adult population when there are insufficient standard doses to protect the population at risk during an outbreak, adding crucial support to WHO policy on the use of fractional dosing of yellow fever vaccine.^5^ The use of fractional dosing could expand the outbreak stockpile up to five times, and therefore will be a crucial back-up tool in case of vaccine supply shortage during yellow fever outbreak response. We are doing substudies to assess immunological non-inferiority and safety of fractional doses among children aged between 9 months and 5 years, and adults living with HIV.

## Data sharing

Data collected for the study, including deidentified participant data, data dictionary and additional related documents such as study protocol and statistical analysis plan, will be made available to others upon request to dpco@epicentre.msf.org, following Epicentre's data sharing policy and in accordance with WHO statement on public disclosure of clinical trial results.
