# Supplementary material for: Immunogenicity and safety of fractional doses of yellow fever vaccines: a randomised, double-blind, non-inferiority trial
Source: Lancet. 2021 Jan 9;397(10269):119–27. doi: 10.1016/S0140-6736(20)32520-4 (PMC7794660; doi:10.1016/S0140-6736(20)32520-4)
Supplement: Supplementary appendix [file mmc1.pdf]

THE LANCET

Supplementary appendix

This appendix formed part of the original submission and has been peer reviewed. We post it as supplied by the authors.

Supplement to: Juan-Giner A, Kimathi D, Grantz KH, et al. Immunogenicity and safety of fractional doses of yellow fever vaccines: a randomised, double-blind, non-inferiority trial. *Lancet* 2021; **397:** 119–27.

Supplementary Appendix

Immunogenicity and safety of fractional doses of yellow fever vaccines: a randomized, double-blind, non-inferiority trial

Aitana Juan-Giner, MSc[1,*], Derick Kimathi, MBChB[2,3*], Kyra H. Grantz, BA[4,5,6], Mainga Hamaluba, MD[2,3], Patrick Kazooba, MD[7], Patricia Njuguna, MMed[2], Gamou Fall, PhD[8], Moussa Dia, MSc[8], Ndeye S. Bob, PhD[8], Thomas P. Monath, MD[9], Alan D. Barrett, PhD[10], Joachim Hombach, PhD[11], Edgar M. Mulogo, PhD[12], Immaculate Ampeire, MBChB[13], Henry K. Karanja, MSc[2], Dan Nyehangane, MSc[7], Juliet Mwanga-Amumpaire, MMed[7], Derek A.T. Cummings, PhD[4,5], Philip Bejon, Prof[2,3], George M. Warimwe, PhD[2,3,#], Rebecca F. Grais, PhD[1,#].

[1] Epicentre, Paris, France
[2] Kenya Medical Research Institute – Wellcome Trust Research Programme, Kilifi, Kenya
[3]Centre for Tropical Medicine & Global Health, University of Oxford, UK
[4] Department of Biology, University of Florida
[5] Emerging Pathogens Institute, University of Florida
[6] Department of Epidemiology, Johns Hopkins Bloomberg School of Public Health
[7] Epicentre Mbarara Research Centre, Uganda
[8] Institut Pasteur Dakar, Senegal
[9] Crozet BioPharma LLC, Devens MA, USA
[10] Sealy Institute for Vaccines Sciences and Department of Pathology, University of Texas Medical Branch, USA
[11] Immunization, Vaccines & Biologicals, World Health Organization
[12] Department of Community Health, Mbarara University of Science & Technology, Uganda
[13] Expanded Program on Immunization, Ministry of Health, Uganda

*Co-first author; #Co-senior author

Corresponding author: Rebecca F Grais; Address: 34 Avenue Jean Jaurès, 75019 Paris, France; Tel: +33 (0) 1 40 21 54 75; Email: rebecca.grais@epicentre.msf.org

Contents

Participants' follow-up 4

Table S1. Summary of early termination by Study Arm, Study Cohort, and Vaccine Manufacturer - Randomized Population 4

Immunogenicity Analysis 5

Immunogenicity assessment 5

Seroconversion and geometric mean titers, per-protocol population 6

Table S2: Non-inferiority of seroconversion and GMT by PRNT90 in fractional vs. standard dose of yellow fever vaccine at Day 10, Day 28 and Day 365 post-vaccination, by vaccine manufacturer, in the per-protocol population 6

Seroconversion, additional analysis 7

Table S3: Non-inferiority of seroconversion rate by $PRNT_{50}$ in fractional vs. standard dose of yellow fever vaccine at Day 28 in intent-to-treat population, by vaccine manufacturer 7

Table S4: Non-inferiority of seroconversion rate by $PRNT_{50}$ in fractional vs. standard dose of yellow fever vaccine at Day 28 in intent-to-treat population with baseline seropositivity to yellow fever, by vaccine manufacturer 7

Geometric mean titers, additional analysis 8

Table S5: Geometric mean titer by $PRNT_{50}$ in fractional vs. standard dose of yellow fever vaccine at Day 28 in intent-to-treat population, by vaccine manufacturer 8

Table S6: Geometric mean titer by $PRNT_{50}$ in fractional vs. standard dose of yellow fever vaccine at Day 28 in intent-to-treat population with baseline seropositivity to yellow fever, by vaccine manufacturer 8

Figure S1: Reverse cumulative distributions of geometric mean titers by $PRNT_{50}$ at Day 28 for per-protocol population, by vaccine manufacturer and by vaccine dose 9

Figure S2: Reverse cumulative distributions of geometric mean titers by PRNT50 at Day 10 for per-protocol population, by vaccine manufacturer and by vaccine dose 10

Figure S3: Reverse cumulative distributions of geometric mean titers by $PRNT_{50}$ at Day 28 for intent-to-treat population with baseline seropositivity to yellow fever, by vaccine manufacturer and by vaccine dose 11

Figure S4: Reverse cumulative distributions of geometric mean titers by $PRNT_{50}$ at Day 10 for intent-to-treat population, by vaccine manufacturer and by vaccine dose 12

Figure S5: Reverse cumulative distributions of geometric mean titers by $PRNT_{50}$ at Day 365 for intent-to-treat population, by vaccine manufacturer and by vaccine dose 13

Figure S6: Geometric mean titer by $PRNT_{50}$ at various timepoints post-vaccination for ITT population, by vaccine manufacturer and by vaccine dose. 14

Geometric mean fold increase 15

Table S7: Geometric mean fold increase by $PRNT_{50}$ in fractional vs. standard dose of yellow fever vaccine at Day 28 in per-protocol population, by vaccine manufacturer 15

Table S8: Geometric mean fold increase by $PRNT_{90}$ in fractional vs. standard dose of yellow fever vaccine at Day 28 in per-protocol population, by vaccine manufacturer 15

Table S9: Geometric mean fold increase by $PRNT_{50}$ in fractional vs. standard dose of yellow fever vaccine at Day 28 in intent-to-treat population, by vaccine manufacturer 15

Table S10: Geometric mean fold increase in by $PRNT_{50}$ fractional vs. standard dose of yellow fever vaccine at Day 28 in intent-to-treat population with baseline seropositivity to yellow fever, by vaccine manufacturer 16

Table S11: Geometric mean fold increase in by $PRNT_{50}$ fractional vs. standard dose of yellow fever vaccine at Day 10, in intent-to-treat population, by vaccine manufacturer 16

Table S12: Geometric mean fold increase in by $PRNT_{50}$ fractional vs. standard dose of yellow fever vaccine at Day 365, in intent-to-treat population, by vaccine manufacturer 16

Safety Analysis 17

Adverse events 17

Table S13: Summary of adverse events up to Day 28 post-vaccination by study arm, and vaccine manufacturer, safety population 17

Table S14: Adverse events up to Day 28 post-vaccination by MedDRA coding (v20.0), study arm and vaccine manufacturer, safety population 17

Table S15: Severity of adverse events up to Day 28 post-vaccination by study arm, and vaccine manufacturer, safety population 20

Table S16: Proportion of participants with at least one related event up to Day 28 post-vaccination, by study arm, and vaccine manufacturer, safety population 20

Table S17: Outcome of adverse events up to Day 28 post-vaccination, by study arm, and vaccine manufacturer, safety population 20

Serious Adverse Events 21

Table S18: Summary of serious adverse events by study arm, and vaccine manufacturer, safety population 21

Table S19: Serious adverse events by MedDRA (v20.0) coding by study arm and vaccine manufacturer, safety population 21

Table S20: Outcome of serious adverse events, by study arm, and vaccine manufacturer, safety population 21

Participants' follow-up

Table S1. Summary of early termination by Study Arm, Study Cohort, and Vaccine Manufacturer - Randomized Population

	Bio-Manguinhos *No. (%)*		Chumakov IPVE *No. (%)*		Institut Pasteur Dakar *No. (%)*		Sanofi Pasteur *No. (%)*	
	Fractional N = 120	Standard N = 120	Fractional N = 120	Standard N = 120	Fractional N = 120	Standard N = 120	Fractional N = 120	Standard N = 120
Total, no. (%)	2 (1.7)	8 (6.7)	5 (4.2)	4 (3.3)	2 (1.7)	5 (4.2)	3 (2.5)	3 (2.5)
SAE	0 (0)	1 (0.8)	0 (0)	0 (0)	1 (0.8)	1 (0.8)	0 (0)	0 (0)
Protocol violation	0 (0)	0 (0)	1 (0.8)	1 (0.8)	0 (0)	0 (0)	0 (0)	0 (0)
Noncompliance with study protocol	1 (0.8)	2 (1.7)	0 (0)	1 (0.8)	0 (0)	0 (0)	0 (0)	0 (0)
Loss to follow-up	1 (0.8)	3 (2.5)	2 (1.7)	0 (0)	1 (0.8)	2 (1.7)	1 (0.8)	0 (0)
Withdrew consent	0 (0)	0 (0)	0 (0)	0 (0)	0 (0)	0 (0)	0 (0)	0 (0)
Moved from study area	0 (0)	2 (1.7)	2 (1.7)	2 (1.7)	0 (0)	2 (1.7)	2 (1.7)	3 (2.5)

Immunogenicity Analysis

Immunogenicity assessment

Viral stock preparation was done in C6/36 cells and titrated by plaque assay method. The attenuated yellow fever vaccine strain 17D is used as challenge strain for the neutralization assays. The yellow fever vaccine strain 17D was provided by the Institut Pasteur de Dakar vaccine production unit (reference 1927). Briefly, the yellow fever vaccine is prepared using various seed strains that were ultimately derived from the 17D strain of yellow fever virus cultured in chicken embryonated eggs. The vaccine was obtained lyophilized. For viral stock preparation, each vial was reconstituted with 5ml of buffer (NaCl 0,9%) and amplified in C636 cells. The passage of the virus stock is 2.

A defined virus concentration of 10^3 plaque forming units (pfu) per ml (dose test, DT) and diluted, heat-inactivated sera (10-fold in Leibovitz's medium containing 3% fetal calf serum, FCS, heated for 20 min at 60°C) were titrated in serial two-fold dilutions from 1:10 to 1:20480. 30 µl of each serum dilution and 30µl of virus were mixed and pre-incubated at 37°C for 1 hour. Calibration range was done with different doses of the challenge virus (DT, 50, 30, 10, 5 and 1% of the DT). Wells with cells alone were used as negative control and wells with undiluted virus as positive controls. In addition, a serum provided by NIBSC was used as international standard for the validation of the calibration range. After 1-hour incubation, 10^6 cells/ml of PS cells in L15-3 % FCS were then added to each well containing the mixture serum/virus and all control wells and incubated for 4 hours at 37°C before overlaying with 0.6% carboxy-methyl-cellulose and 3% FCS in L-15 Leibovitz's media. Plates were sealed and incubated for 4 to 5 days at 37°C for the development of cytopathic effect. Wells were washed in phosphate buffer saline and stained in amino black solution (0.1%) for 30 minutes at room temperature. Plates were rinsed with water and allowed to dry then infectious plaques visualized.

The test was considered valid if: (1) there was no lysis plaques in the negative control wells; (2) total lysis in the undiluted virus wells; and, (3) linear lysis range in wells containing the different doses of the challenge virus (30-50 lysis plaques for the DT, 15-25 lysis plaques for the half dose and 3-5 lysis plaques for the 10% dose). Inhibition percentage of the DT for a serum was then obtained by comparing the results with the calibration range. Plaques were counted for each serum and antibody titer was determined as the antibody dilution that reduced observed lysis plaques by 90% and 50% compared to the number observed in the calibration range ($PRNT_{90}$ and $PRNT_{50}$).

Seroconversion and geometric mean titers, per-protocol population

Table S2: Non-inferiority of seroconversion and GMT by PRNT90 in fractional vs. standard dose of yellow fever vaccine at Day 10, Day 28 and Day 365 post-vaccination, by vaccine manufacturer, in the per-protocol population

Manufacturer	Time	Dosage	N	Seroconversion			GMT	
				n	% (95% CI)	Difference, % (95% CI)	GMT (95% CI)	Ratio GMT (95% CI)
Bio-Manguinhos	D10	Fractional	110	8	7.3 (3.2, 13.8)	-2.13 (-9.74, 5.41)	6.6 (5.9, 7.4)	1.00 (0.85, 1.17)
		Standard	117	11	9.4 (4.8, 16.2)		6.6 (5.9, 7.4)	
	D28	Fractional	111	110	99.1 (95.1, 100.0)	3.37 (-2.02, 8.08)	379 (282, 509)	0.89 (0.58, 1.34)
		Standard	117	112	95.7 (90.3, 98.6)		428 (318, 575)	
	D365	Fractional	110	102	92.7 (86.2, 96.8)	6.24 (-2.14, 14.5)	95.4 (73.3, 124)	1.22 (0.84, 1.79)
		Standard	111	96	86.5 (78.7, 92.2)		78.0 (59.3, 103)	
Chumakov IPVE	D10	Fractional	111	8	7.2 (3.2, 13.7)	-10.34 (-19.01, -1.59)	6.3 (5.7, 6.9)	0.83 (0.71, 0.97)
		Standard	114	20	17.5 (11.1, 25.8)		7.5 (6.6, 8.5)	
	D28	Fractional	111	105	94.6 (88.6, 98.0)	-2.77 (-8.40, 3.36)	561 (399,789)	1.14 (0.74, 1.76)
		Standard	114	111	97.4 (92.5, 99.5)		493 (375, 648)	
	D365	Fractional	107	98	91.6 (84.6, 96.1)	-2.16 (-9.41, 5.35)	125.9 (94.4, 168)	1.40 (0.96, 2.03)
		Standard	112	105	93.8 (87.5, 97.5)		90.0 (70.9, 114)	
Institut Pasteur Dakar	D10	Fractional	112	16	14.3 (8.4, 22.2)	-14.42 (-25.27, -3.85)	7.2 (6.4, 8.2)	0.74 (0.60, 0.92)
		Standard	108	31	28.7 (20.4, 38.2)		9.7 (8.2, 11.4)	
	D28	Fractional	112	111	99.1 (95.1, 100.0)	3.65 (-2.05, 8.41)	376 (294, 481)	1.35 (0.92, 1.98)
		Standard	110	105	95.5 (89.7, 98.5)		279 (208, 374)	
	D365	Fractional	111	105	94.6 (88.6, 98.0)	0.25 (-6.54, 6.88)	106 (82.7, 136)	1.40 (0.99, 1.98)
		Standard	106	100	94.3 (88.1, 97.9)		75.9 (59.3, 97.1)	
Sanofi Pasteur	D10	Fractional	113	14	12.4 (6.9, 19.9)	-18.24 (-28.96, -7.92)	6.9 (6.2, 7.7)	0.71 (0.59, 0.86)
		Standard	111	34	30.6 (22.2, 40.1)		9.7 (8.3, 11.3)	
	D28	Fractional	113	110	97.3 (92.4, 99.5)	-1.76 (-6.12, 3.15)	498 (385, 644)	1.21 (0.84, 1.76)
		Standard	112	111	99.1 (95.1, 100.0)		410 (312, 538)	
	D365	Fractional	109	104	95.4 (89.6, 98.5)	-0.92 (-6.91, 5.21)	95.6 (76.7, 119)	0.82 (0.58, 1.14)
		Standard	109	105	96.3 (90.9, 99.0)		117 (90.3, 152)	

Seroconversion, additional analysis

Table S3: Non-inferiority of seroconversion rate by $PRNT_{50}$ in fractional vs. standard dose of yellow fever vaccine at Day 28 in intent-to-treat population, by vaccine manufacturer

**Manufacturer**	**Fractional dose**		**Standard dose**		**Difference (Fractional - Standard)**
	No. SC	% SC (95% CI)	No. SC	% SC (95% CI)	(95% CI)
Bio-Manguinhos	117/117	100.0 (96.9, 100.0)	116/118	98.3 (94.0, 99.8)	1.69 (-2.58, 5.10)
Chumakov IPVE	115/118	97.5 (92.7, 99.5)	118/118	100.0 (96.9, 100.0)	-2.54 (-6.11, 2.12)
Institut Pasteur Dakar	119/119	100.0 (96.9, 100.0)	116/119	97.5 (92.8, 99.5)	2.52 (-2.11, 6.06)
Sanofi Pasteur	120/120	100.0 (97.0, 100.0)	120/120	100.0 (97.0, 100.0)	0.0 (-3.10, 3.10)

Table S4: Non-inferiority of seroconversion rate by $PRNT_{50}$ in fractional vs. standard dose of yellow fever vaccine at Day 28 in intent-to-treat population with baseline seropositivity to yellow fever, by vaccine manufacturer

**Manufacturer**	**Fractional dose**		**Standard dose**		**Difference (Fractional - Standard)**
	No. SC	% SC (95% CI)	No. SC	% SC (95% CI)	(95% CI)
Bio-Manguinhos	6/6	100.0 (54.1, 100.0)	1/1	100.0 (2.5, 100.0)	0.0 (-79.3, 39.0)
Chumakov IPVE	5/7	71.4 (29.0, 96.3)	4/4	100.0 (39.8, 100.0)	-28.57 (-81.6, 7.0)
Institut Pasteur Dakar	7/7	100.0 (59.0, 100.0)	8/9	88.9 (51.8, 99.7)	11.1 (-21.3, 47.7)
Sanofi Pasteur	7/7	100.0 (59.0, 100.0)	8/8	100.0 (63.1, 100.0)	0.0 (-32.4, 35.4)

Geometric mean titers, additional analysis

Table S5: Geometric mean titer by $PRNT_{50}$ in fractional vs. standard dose of yellow fever vaccine at Day 28 in intent-to-treat population, by vaccine manufacturer

Manufacturer	Fractional dose			Standard dose			Difference - log GMT (Fractional – Standard)	Ratio - GMT (Fractional/ Standard)
	N	GMT	95% CI	N	GMT	95% CI	(95% CI)	(95% CI)
Bio-Manguinhos	117	3718.2	2676.0, 5166.3	118	4119.8	2895.4, 5862.1	-0.045 (-0.253, 0.164)	0.90 (0.56, 1.46)
Chumakov IPVE	118	5826.3	4152.9, 8174.1	118	5493.9	4045.0, 7461.9	0.026 (-0.172, 0.223)	1.06 (0.67, 1.67)
Institut Pasteur Dakar	119	4224.7	3170.7, 5629.0	119	2530.4	1787.6, 3581.8	0.223 (0.028, 0.417)	1.67 (1.07, 2.61)
Sanofi Pasteur	120	5713.9	4290.7, 7609.1	120	4280.6	3105.3, 5900.7	0.125 (-0.060, 0.311)	1.33 (0.87, 2.05)

Table S6: Geometric mean titer by $PRNT_{50}$ in fractional vs. standard dose of yellow fever vaccine at Day 28 in intent-to-treat population with baseline seropositivity to yellow fever, by vaccine manufacturer

Manufacturer	Fractional dose			Standard dose			Difference - log GMT (Fractional – Standard)	Ratio - GMT (Fractional/ Standard)
	N	GMT	95% CI	N	GMT	95% CI	(95% CI)	(95% CI)
Bio-Manguinhos	6	1280.0	215.5, 7603.8	1	20480	--	-1.204 (-3.251, 0.843)	0.063 (0.001, 6.970)
Chumakov IPVE	7	5120.0	555.7, 47173.5	4	1076.3	22.7, 51106.1	0.667 (-0.914, 2.268)	4.76 (0.12, 185.45)
Institut Pasteur Dakar	7	3445.5	721.1, 16462.5	9	2031.9	522.3, 7903.7	0.229 (-0.584, 1.043)	1.70 (0.26, 11.04)
Sanofi Pasteur	7	9274.6	3628.7, 23705.0	8	4695.1	1447.1, 15232.8	0.296 (-0.296, 0.887)	1.98 (0.51, 7.71)

Figure S1: Reverse cumulative distributions of geometric mean titers by $PRNT_{50}$ at Day 28 for per-protocol population, by vaccine manufacturer and by vaccine dose. (IPD: Institut Pasteur Dakar)

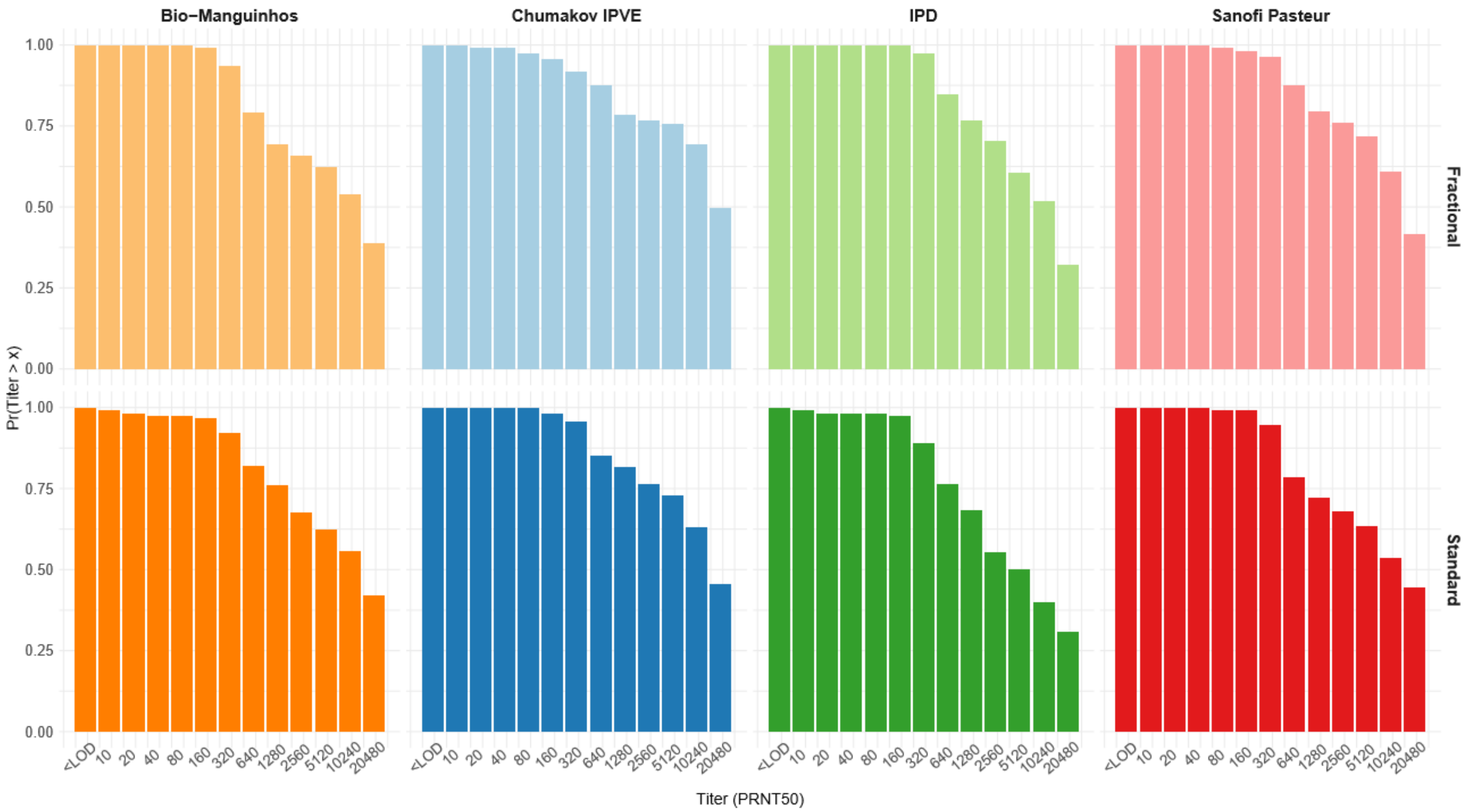

Figure S2: Reverse cumulative distributions of geometric mean titers by PRNT50 at Day 10 for per-protocol population, by vaccine manufacturer and by vaccine dose. (IPD: Institut Pasteur Dakar)

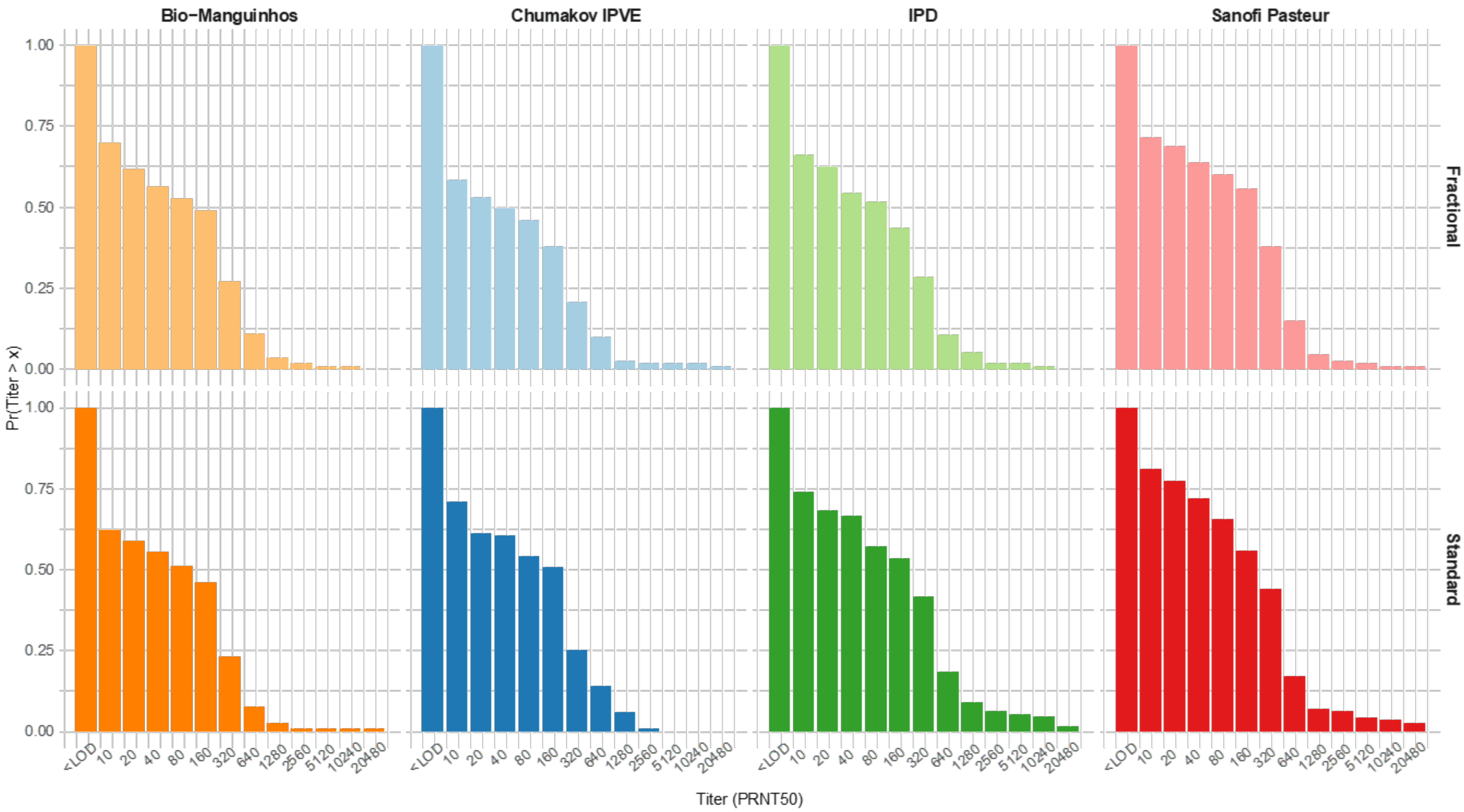

Figure S3: Reverse cumulative distributions of geometric mean titers by $PRNT_{50}$ at Day 28 for intent-to-treat population with baseline seropositivity to yellow fever, by vaccine manufacturer and by vaccine dose. (IPD: Institut Pasteur Dakar)

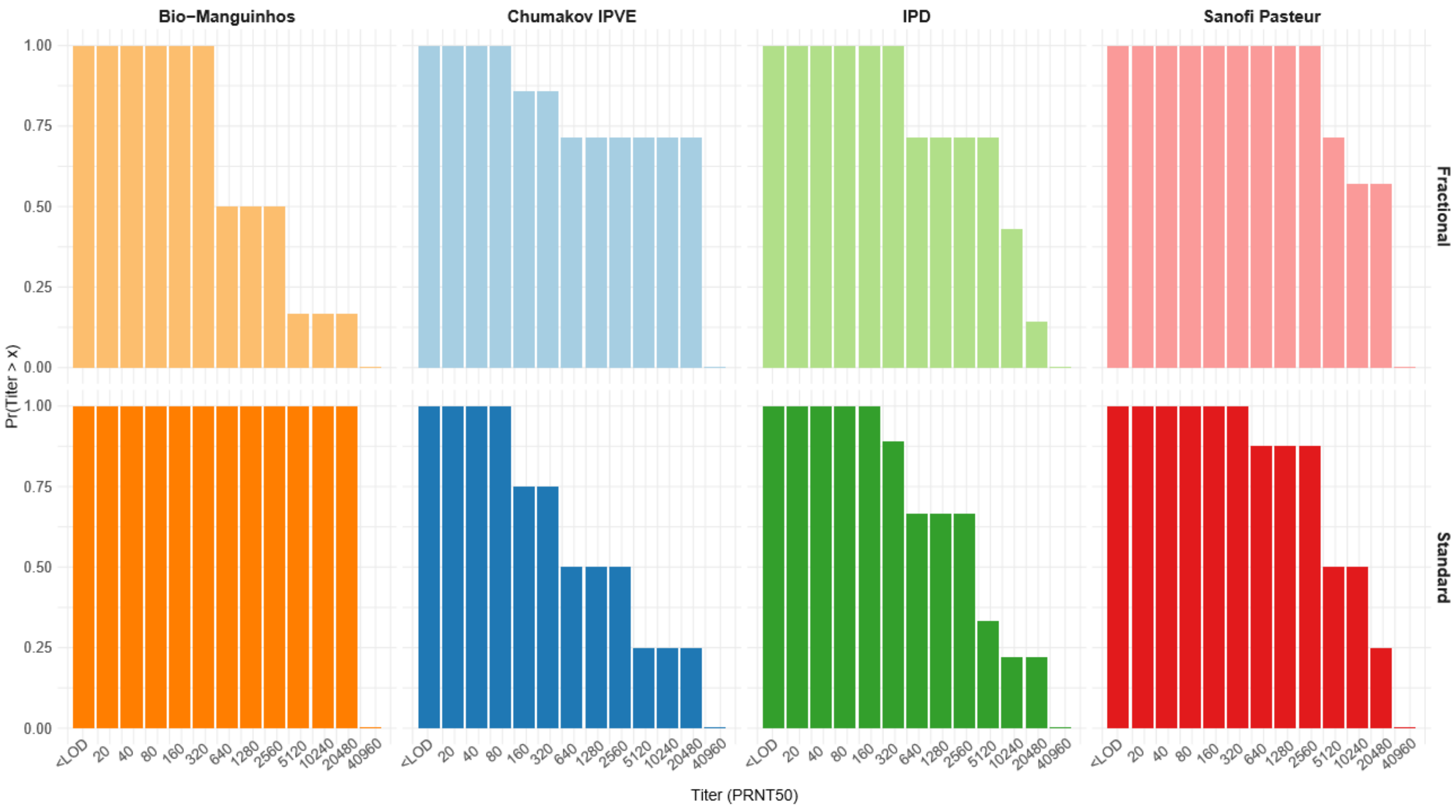

Figure S4: Reverse cumulative distributions of geometric mean titers by $PRNT_{50}$ at Day 10 for intent-to-treat population, by vaccine manufacturer and by vaccine dose. (IPD: Institut Pasteur Dakar)

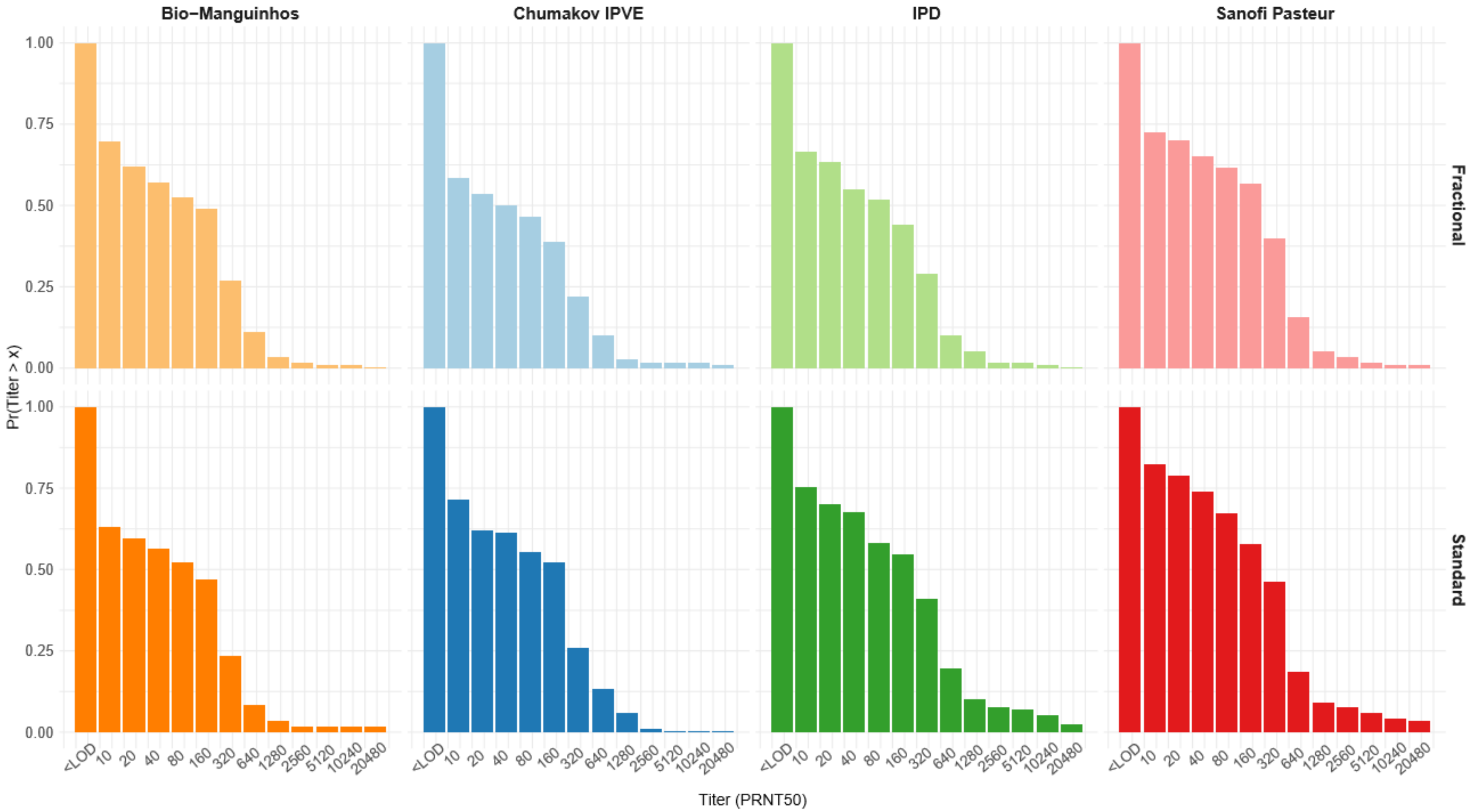

Figure S5: Reverse cumulative distributions of geometric mean titers by $PRNT_{50}$ at Day 365 for intent-to-treat population, by vaccine manufacturer and by vaccine dose. (IPD: Institut Pasteur Dakar)

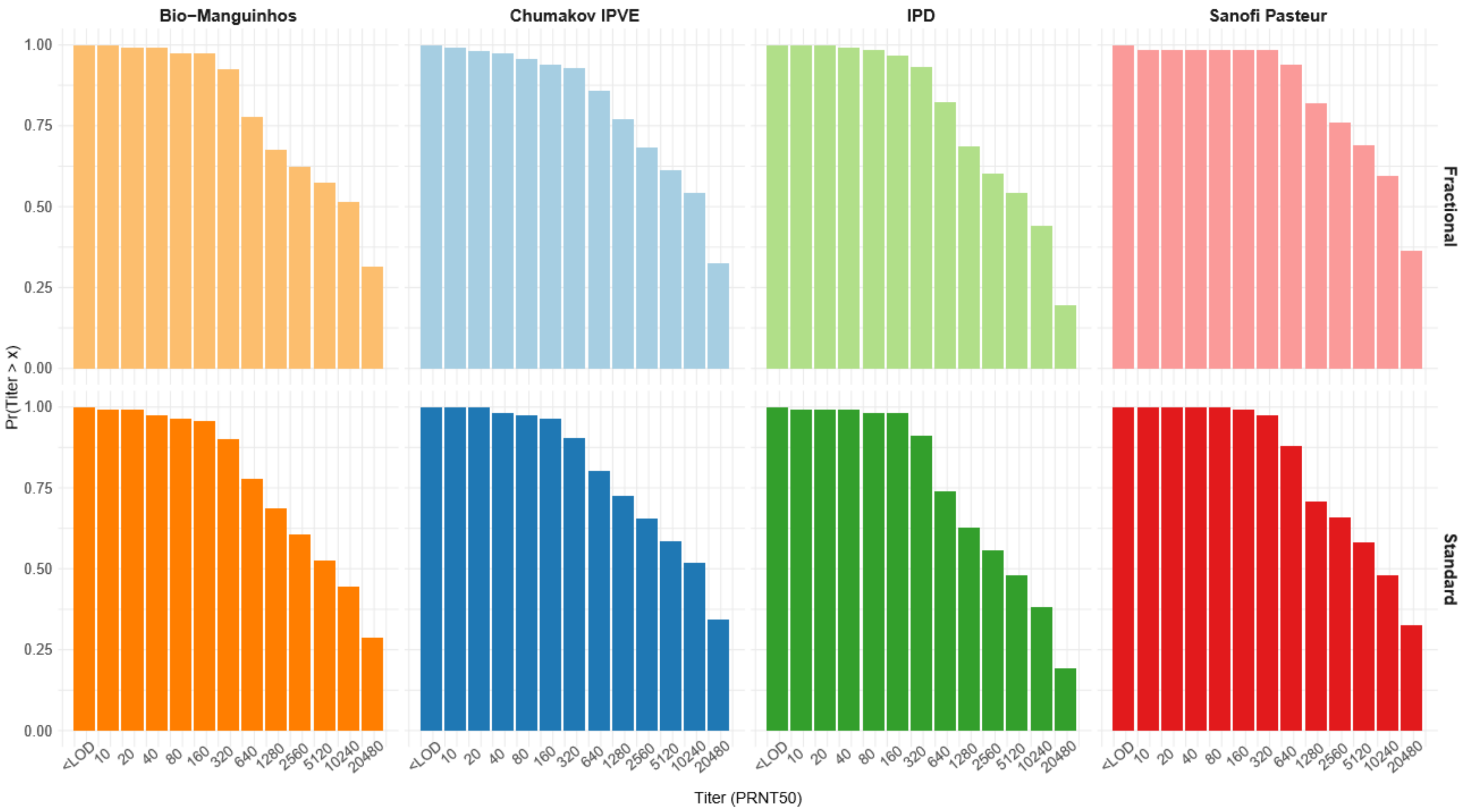

Figure S6: Geometric mean titer by $PRNT_{50}$ at various timepoints post-vaccination for ITT population, by vaccine manufacturer and by vaccine dose. (IPD: Institut Pasteur Dakar)

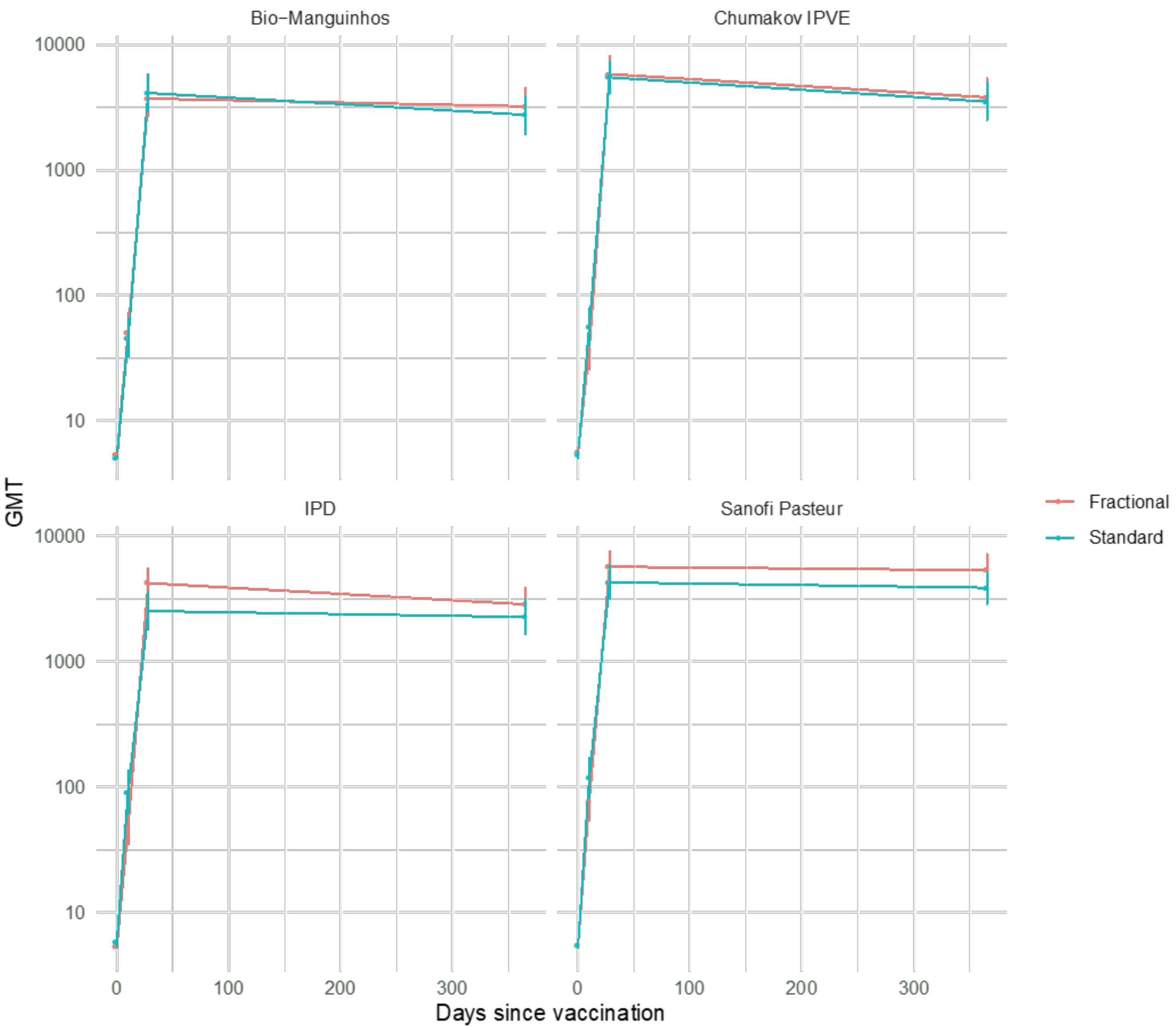

Note: vertical bars represent 95% confidence intervals.

Geometric mean fold increase

Table S7: Geometric mean fold increase by $PRNT_{50}$ in fractional vs. standard dose of yellow fever vaccine at Day 28 in per-protocol population, by vaccine manufacturer

Manufacturer	Fractional dose			Standard dose			Difference - log GMFI (Fractional – Standard)	Ratio - GMFI (Fractional/ Standard)
	N	GMFI	95% CI	N	GMFI	95% CI	(95% CI)	(95% CI)
Bio-Manguinhos	111	788	562, 1103	117	813	570, 1159	-0.014 (-0.225, 0.198)	0.97 (0.60, 1.58)
Chumakov IPVE	111	1175	832, 1658	114	1163	859.3, 1575	0.004 (-0.194, 0.202)	1.01 (0.64, 1.59)
Institut Pasteur Dakar	112	856	636, 1151	110	515	358, 742	0.220 (0.017, 0.423)	1.66 (1.04, 2.65)
Sanofi Pasteur	113	1109	821, 1498	112	850	607, 1192	0.115 (-0.080, 0.311)	1.30 (0.83, 2.04)

Table S8: Geometric mean fold increase by $PRNT_{90}$ in fractional vs. standard dose of yellow fever vaccine at Day 28 in per-protocol population, by vaccine manufacturer

Manufacturer	Fractional dose			Standard dose			Difference - log GMFI (Fractional – Standard)	Ratio - GMFI (Fractional/ Standard)
	N	GMFI	95% CI	N	GMFI	95% CI	(95% CI)	(95% CI)
Bio-Manguinhos	111	75.8	56.4, 101.8	117	85.6	63.6, 115.1	-0.053 (-0.234, 0.128)	0.89 (0.58, 1.34)
Chumakov IPVE	111	112.3	79.9, 157.8	114	98.6	75.0, 129.5	0.057 (-0.132, 0.245)	1.14 (0.74, 1.76)
Institut Pasteur Dakar	112	75.2	58.8, 96.1	110	55.7	41.5, 74.8	0.130 (-0.036, 0.296)	1.35 (0.92, 1.98)
Sanofi Pasteur	113	99.5	76.9, 128.8	112	82.0	62.4, 107.7	0.084 (-0.078, 0.246)	1.21 (0.84, 1.76)

Table S9: Geometric mean fold increase by $PRNT_{50}$ in fractional vs. standard dose of yellow fever vaccine at Day 28 in intent-to-treat population, by vaccine manufacturer

Manufacturer	Fractional dose			Standard dose			Difference - log GMFI (Fractional – Standard)	Ratio - GMFI (Fractional/ Standard)
	N	GMFI	95% CI	N	GMFI	95% CI	(95% CI)	(95% CI)
Bio-Manguinhos	117	696.7	495.7, 979.4	118	809.6	569.5, 1150.8	-0.065 (-0.277, 0.146)	0.86 (0.53, 1.40)
Chumakov IPVE	118	1036.1	716.6, 1498.1	118	1018.0	732.6, 1414.6	0.008 (-0.206, 0.221)	1.02 (0.62, 1.66)
Institut Pasteur Dakar	119	787.9	588.0, 1055.7	119	432.4	299.8, 623.7	0.261 (0.058, 0.463)	1.82 (1.14, 2.91)
Sanofi Pasteur	120	1047.9	780.5, 1406.9	120	789.6	571.0, 1091.8	0.123 (-0.066, 0.312)	1.33 (0.86, 2.05)

Table S10: Geometric mean fold increase in by $PRNT_{50}$ fractional vs. standard dose of yellow fever vaccine at Day 28 in intent-to-treat population with baseline seropositivity to yellow fever, by vaccine manufacturer

**Manufacturer**	**Fractional dose**			**Standard dose**			**Difference - log GMFI (Fractional – Standard)**	**Ratio - GMFI (Fractional/ Standard)**
	N	GMFI	95% CI	N	GMFI	95% CI	(95% CI)	(95% CI)
Bio-Manguinhos	6	71.8	11.1, 463.3	1	512	--	-0.853 (-2.995, 1.289)	0.14 (0.001, 19.5)
Chumakov IPVE	7	141.3	4.8, 4153.2	4	22.6	0.4, 1334.9	0.796 (-1.077, 2.668)	6.25 (0.08, 465.7)
Institut Pasteur Dakar	7	210.0	46.2, 953.6	9	50.8	11.8, 218.5	0.616 (-0.209, 1.442)	4.13 (0.62, 27.6)
Sanofi Pasteur	7	420.0	81.2, 2172.5	8	279.2	93.6, 832.3	0.177 (-0.602, 0.957)	1.50 (0.25, 9.06)

Table S11: Geometric mean fold increase in by $PRNT_{50}$ fractional vs. standard dose of yellow fever vaccine at Day 10, in intent-to-treat population, by vaccine manufacturer

**Manufacturer**	**Fractional dose**			**Standard dose**			**Difference - log GMFI (Fractional – Standard)**	**Ratio - GMFI (Fractional/ Standard)**
	N	GMFI	95% CI	N	GMFI	95% CI	(95% CI)	(95% CI)
Bio-Manguinhos	116	9.7	6.8, 14.0	119	9.0	6.3, 12.9	0.033 (-0.187, 0.252)	1.08 (0.65, 1.79)
Chumakov IPVE	118	6.6	4.5, 9.5	119	10.4	7.3, 14.9	-0.201 (-0.424, 0.022)	0.63 (0.38, 1.05)
Institut Pasteur Dakar	120	9.1	6.3, 13.2	117	15.5	10.3, 23.3	-0.230 (-0.468, 0.007)	0.59 (0.34, 1.02)
Sanofi Pasteur	120	14.0	9.7, 20.2	119	21.7	14.9, 31.4	-0.189 (-0.414, 0.036)	0.65 (0.39, 1.09)

Table S12: Geometric mean fold increase in by $PRNT_{50}$ fractional vs. standard dose of yellow fever vaccine at Day 365, in intent-to-treat population, by vaccine manufacturer

**Manufacturer**	**Fractional dose**			**Standard dose**			**Difference - log GMFI (Fractional – Standard)**	**Ratio - GMFI (Fractional/ Standard)**
	N	GMFI	95% CI	N	GMFI	95% CI	(95% CI)	(95% CI)
Bio-Manguinhos	117	604	423, 864	112	541	376, 779	0.048 (-0.172, 0.268)	1.12 (0.67, 1.85)
Chumakov IPVE	114	673	456, 995	116	650	449, 941	0.015 (-0.217, 0.247)	1.04 (0.61, 1.77)
Institut Pasteur Dakar	118	533	386, 738	115	386	272, 547	0.141 (-0.065, 0.347)	1.38 (0.86, 2.22)
Sanofi Pasteur	116	976	722, 1320	117	713	525, 969	0.136 (-0.049, 0.322)	1.37 (0.89, 2.10)

Safety Analysis

Adverse events

Table S13: Summary of adverse events up to Day 28 post-vaccination by study arm, and vaccine manufacturer, safety population

Manufacturer	Fractional Dose		Standard Dose		P value
	N	No. with ≥1 AE (%)	N	No. with ≥1 AE (%)	
Bio-Manguinhos	120	58 (48.3)	120	69 (57.5)	0.20
Chumakov IPVE	119	69 (58.0)	120	72 (60.0)	0.85
Institut Pasteur Dakar	120	76 (63.3)	120	72 (60.0)	0.69
Sanofi Pasteur	120	60 (50.0)	120	70 (53.8)	0.24

Table S14: Adverse events up to Day 28 post-vaccination by MedDRA coding (v20.0), study arm and vaccine manufacturer, safety population

System Organ Class and Preferred Term	Bio-Manguinhos		Chumakov IPVE		Institut Pasteur Dakar		Sanofi Pasteur	
n (%) with ≥ 1 AE	Frac. N=120	Standard N=120	Frac. N=119	Standard N=120	Frac. N=120	Standard N=120	Frac. N=120	Standard N=120
**Blood and lymphatic system disorders**	0 (0)	0 (0)	0 (0)	0 (0)	0 (0)	0 (0)	1 (0.8)	1 (0.8)
Anemia	0 (0)	0 (0)	0 (0)	0 (0)	0 (0)	0 (0)	1 (0.8)	0 (0)
Lymphadenopathy	0 (0)	0 (0)	0 (0)	0 (0)	0 (0)	0 (0)	0 (0)	1 (0.8)
**Cardiac disorders**	0 (0)	0 (0)	1 (0.8)	0 (0)	0 (0)	0 (0)	0 (0)	0 (0)
Palpitations	0 (0)	0 (0)	1 (0.8)	0 (0)	0 (0)	0 (0)	0 (0)	0 (0)
**Ear and labyrinth disorders**	1 (0.8)	0 (0)	0 (0)	0 (0)	1 (0.8)	1 (0.8)	0 (0)	1 (0.8)
Allergic Otitis Externa	0 (0)	0 (0)	0 (0)	0 (0)	0 (0)	0 (0)	0 (0)	1 (0.8)
Ear Pain	0 (0)	0 (0)	0 (0)	0 (0)	0 (0)	1 (0.8)	0 (0)	0 (0)
Sudden Hearing Loss	1 (0.8)	0 (0)	0 (0)	0 (0)	1 (0.8)	0 (0)	0 (0)	0 (0)
**Eye disorders**	0 (0)	0 (0)	2 (1.7)	1 (0.8)	2 (1.7)	0 (0)	0 (0)	0 (0)
Conjunctivitis Allergic	0 (0)	0 (0)	2 (1.7)	0 (0)	2 (1.7)	0 (0)	0 (0)	0 (0)
Eye Pruritus	0 (0)	0 (0)	0 (0)	1 (0.8)	0 (0)	0 (0)	0 (0)	0 (0)
**Gastrointestinal disorders**	8 (6.7)	14 (11.7)	15 (12.6)	15 (12.5)	13 (10.8)	12 (10.0)	11 (9.2)	13 (10.8)
Abdominal discomfort	0 (0)	0 (0)	1 (0.8)	0 (0)	0 (0)	0 (0)	0 (0)	0 (0)
Abdominal distension	0 (0)	0 (0)	0 (0)	0 (0)	0 (0)	0 (0)	1 (0.8)	0 (0)
Abdominal pain	4 (3.3)	3 (2.5)	3 (2.5)	8 (6.7)	9 (7.5)	1 (0.8)	6 (5.0)	3 (2.5)
Diarrhea	2 (1.7)	2 (1.7)	4 (3.4)	2 (1.7)	0 (0)	5 (4.2)	0 (0)	0 (0)
Dyspepsia	0 (0)	0 (0)	0 (0)	0 (0)	0 (0)	1 (0.8)	0 (0)	0 (0)
Epigastric discomfort	0 (0)	0 (0)	1 (0.8)	0 (0)	0 (0)	0 (0)	0 (0)	0 (0)
Food poisoning	0 (0)	0 (0)	0 (0)	0 (0)	0 (0)	0 (0)	0 (0)	1 (0.8)
Gastritis	0 (0)	1 (0.8)	2 (1.7)	1 (0.8)	2 (1.7)	0 (0)	1 (0.8)	2 (1.7)
Gastrointestinal disorder	2 (1.7)	0 (0)	0 (0)	1 (0.8)	1 (0.8)	2 (1.7)	1 (0.8)	0 (0)
Gastroesophageal reflux disease	0 (0)	1 (0.8)	0 (0)	0 (0)	0 (0)	0 (0)	0 (0)	0 (0)
Nausea	1 (0.8)	6 (5.0)	3 (2.5)	2 (1.7)	2 (1.7)	3 (2.5)	2 (1.7)	7 (5.8)
Peptic ulcer	0 (0)	0 (0)	1 (0.8)	0 (0)	0 (0)	0 (0)	0 (0)	1 (0.8)
Stomatitis	0 (0)	0 (0)	0 (0)	1 (0.8)	0 (0)	0 (0)	0 (0)	0 (0)
Toothache	0 (0)	1 (0.8)	0 (0)	0 (0)	0 (0)	0 (0)	0 (0)	0 (0)
Vomiting	0 (0)	0 (0)	1 (0.8)	0 (0)	0 (0)	0 (0)	1 (0.8)	0 (0)

**General disorders and administration site conditions**	19 (15.8)	25 (20.8)	29 (24.4)	21 (17.5)	29 (24.2)	25 (20.8)	30 (25.0)	25 (20.8)
Chest pain	1 (0.8)	0 (0)	0 (0)	1 (0.8)	0 (0)	0 (0)	0 (0)	0 (0)
Fatigue	8 (6.7)	15 (12.5)	18 (15.1)	12 (10.0)	19 (15.8)	21 (17.5)	21 (17.5)	17 (14.2)
Injection site discomfort	0 (0)	0 (0)	0 (0)	1 (0.8)	0 (0)	0 (0)	0 (0)	0 (0)
Injection site hypoesthesia	0 (0)	0 (0)	0 (0)	0 (0)	0 (0)	0 (0)	1 (0.8)	0 (0)
Injection site reaction	1 (0.8)	0 (0)	0 (0)	0 (0)	0 (0)	0 (0)	0 (0)	0 (0)
Local reaction	1 (0.8)	1 (0.8)	1 (0.8)	1 (0.8)	0 (0)	0 (0)	0 (0)	1 (0.8)
Malaise	0 (0)	0 (0)	0 (0)	1 (0.8)	0 (0)	0 (0)	0 (0)	0 (0)
Pyrexia	9 (7.5)	11 (9.2)	12 (10.1)	9 (7.5)	12 (10.0)	9 (7.5)	10 (8.3)	14 (11.7)
Vaccination site discomfort	0 (0)	1 (0.8)	0 (0)	1 (0.8)	1 (0.8)	0 (0)	0 (0)	0 (0)
Vaccination site irritation	0 (0)	1 (0.8)	0 (0)	0 (0)	0 (0)	0 (0)	0 (0)	0 (0)
Vaccination site pain	1 (0.8)	0 (0)	0 (0)	0 (0)	0 (0)	0 (0)	1 (0.8)	0 (0)
Vaccination site paresthesia	0 (0)	0 (0)	0 (0)	0 (0)	0 (0)	0 (0)	1 (0.8)	0 (0)
**Infections and infestations**	16 (13.3)	19 (15.8)	14 (11.8)	15 (12.5)	20 (16.7)	18 (15.0)	9 (7.5)	15 (12.5)
Abscess	0 (0)	1 (0.8)	0 (0)	0 (0)	0 (0)	0 (0)	0 (0)	1 (0.8)
Acrodermatitis	0 (0)	0 (0)	1 (0.8)	0 (0)	0 (0)	0 (0)	0 (0)	0 (0)
Bacterial vaginosis	1 (0.8)	0 (0)	0 (0)	0 (0)	1 (0.8)	1 (0.8)	1 (0.8)	0 (0)
Dysentery	0 (0)	1 (0.8)	0 (0)	0 (0)	0 (0)	0 (0)	0 (0)	0 (0)
Eye infection, bacterial	0 (0)	0 (0)	0 (0)	0 (0)	0 (0)	0 (0)	1 (0.8)	0 (0)
Folliculitis	0 (0)	0 (0)	0 (0)	1 (0.8)	0 (0)	0 (0)	0 (0)	0 (0)
Fungal skin infection	1 (0.8)	0 (0)	0 (0)	1 (0.8)	0 (0)	0 (0)	0 (0)	0 (0)
Gastroenteritis	0 (0)	0 (0)	0 (0)	2 (1.7)	0 (0)	2 (1.7)	1 (0.8)	1 (0.8)
Genitourinary tract infection	0 (0)	0 (0)	0 (0)	1 (0.8)	0 (0)	0 (0)	0 (0)	0 (0)
Lower respiratory tract infection	0 (0)	0 (0)	0 (0)	1 (0.8)	1 (0.8)	0 (0)	0 (0)	0 (0)
Mastitis	0 (0)	0 (0)	0 (0)	0 (0)	0 (0)	1 (0.8)	0 (0)	1 (0.8)
Orchitis	0 (0)	0 (0)	0 (0)	1 (0.8)	0 (0)	0 (0)	0 (0)	0 (0)
Pelvic inflammatory disease	0 (0)	2 (1.7)	1 (0.8)	1 (0.8)	0 (0)	0 (0)	0 (0)	1 (0.8)
Pharyngitis	0 (0)	1 (0.8)	1 (0.8)	0 (0)	2 (1.7)	0 (0)	1 (0.8)	1 (0.8)
Respiratory tract infection	3 (2.5)	2 (1.7)	4 (3.4)	6 (5.0)	6 (5.0)	2 (1.7)	1 (0.8)	3 (2.5)
Respiratory tract infection, viral	0 (0)	0 (0)	1 (0.8)	0 (0)	0 (0)	0 (0)	0 (0)	0 (0)
Rhinitis	0 (0)	4 (3.3)	1 (0.8)	3 (2.5)	3 (2.5)	2 (1.7)	0 (0)	2 (1.7)
Skin bacterial infection	0 (0)	0 (0)	1 (0.8)	0 (0)	0 (0)	0 (0)	0 (0)	0 (0)
Tinea capitis	0 (0)	0 (0)	1 (0.8)	0 (0)	0 (0)	1 (0.8)	0 (0)	0 (0)
Tonsillitis	1 (0.8)	1 (0.8)	1 (0.8)	0 (0)	1 (0.8)	2 (1.7)	2 (1.7)	1 (0.8)
Upper respiratory tract infection	4 (3.3)	3 (2.5)	0 (0)	0 (0)	4 (3.3)	5 (4.2)	1 (0.8)	0 (0)
Urinary tract infection	5 (4.2)	2 (1.7)	3 (2.5)	0 (0)	2 (1.7)	2 (1.7)	1 (0.8)	1 (0.8)
Viral rhinitis	1 (0.8)	0 (0)	1 (0.8)	0 (0)	0 (0)	0 (0)	0 (0)	1 (0.8)
Viral upper respiratory tract infection	0 (0)	0 (0)	0 (0)	0 (0)	1 (0.8)	0 (0)	0 (0)	0 (0)
Vulvovaginal candidiasis	0 (0)	3 (2.5)	0 (0)	0 (0)	0 (0)	2 (1.7)	0 (0)	3 (2.5)
Wound sepsis	0 (0)	0 (0)	0 (0)	0 (0)	0 (0)	1 (0.8)	1 (0.8)	0 (0)
**Injury, poisoning, and procedural complaints**	0 (0)	0 (0)	0 (0)	0 (0)	0 (0)	0 (0)	0 (0)	2 (1.7)
Injury	0 (0)	0 (0)	0 (0)	0 (0)	0 (0)	0 (0)	0 (0)	1 (0.8)

Road traffic accident	0 (0)	0 (0)	0 (0)	0 (0)	0 (0)	0 (0)	0 (0)	1 (0.8)
**Metabolism and nutrition disorders**	1 (0.8)	0 (0)	0 (0)	0 (0)	0 (0)	1 (0.8)	0 (0)	3 (2.5)
Abnormal loss of weight	1 (0.8)	0 (0)	0 (0)	0 (0)	0 (0)	0 (0)	0 (0)	0 (0)
Decreased appetite	0 (0)	0 (0)	0 (0)	0 (0)	0 (0)	1 (0.8)	0 (0)	3 (2.5)
**Musculoskeletal and connective tissue disorders**	20 (16.7)	22 (18.3)	20 (16.8)	19 (15.8)	21 (17.5)	26 (21.7)	19 (15.8)	19 (15.8)
Arthralgia	4 (3.3)	3 (2.5)	4 (3.4)	2 (1.7)	4 (3.3)	2 (1.7)	4 (3.3)	2 (1.7)
Back pain	1 (0.8)	1 (0.8)	2 (1.7)	2 (1.7)	4 (3.3)	2 (1.7)	1 (0.8)	1 (0.8)
Musculoskeletal chest pain	1 (0.8)	0 (0)	0 (0)	0 (0)	0 (0)	0 (0)	0 (0)	0 (0)
Musculoskeletal pain	1 (0.8)	1 (0.8)	2 (1.7)	1 (0.8)	0 (0)	0 (0)	1 (0.8)	0 (0)
Myalgia	15 (12.5)	18 (15.0)	14 (11.8)	15 (12.5)	14 (11.7)	22 (18.3)	14 (11.7)	16 (13.3)
**Nervous system disorders**	24 (20.0)	29 (24.2)	30 (25.2)	36 (30.0)	33 (27.5)	25 (20.8)	30 (25.0)	29 (24.2)
Dizziness	6 (5.0)	4 (3.3)	3 (2.5)	4 (3.3)	4 (3.3)	5 (4.2)	3 (2.5)	2 (1.7)
Headache	21 (17.5)	26 (21.7)	27 (22.7)	30 (25.0)	30 (25.0)	22 (18.3)	29 (24.2)	28 (23.3)
Hypoesthesia	0 (0)	0 (0)	0 (0)	0 (0)	0 (0)	0 (0)	1 (0.8)	0 (0)
Neuropathy peripheral	0 (0)	0 (0)	0 (0)	1 (0.8)	0 (0)	0 (0)	0 (0)	0 (0)
Paresthesia	0 (0)	1 (0.8)	0 (0)	1 (0.8)	0 (0)	1 (0.8)	0 (0)	0 (0)
Somnolence	0 (0)	1 (0.8)	0 (0)	0 (0)	0 (0)	0 (0)	0 (0)	0 (0)
**Psychiatric disorders**	0 (0)	0 (0)	0 (0)	0 (0)	0 (0)	0 (0)	2 (1.7)	0 (0)
Insomnia	0 (0)	0 (0)	0 (0)	0 (0)	0 (0)	0 (0)	1 (0.8)	0 (0)
Libido decreased	0 (0)	0 (0)	0 (0)	0 (0)	0 (0)	0 (0)	1 (0.8)	0 (0)
**Renal and urinary disorders**	0 (0)	0 (0)	0 (0)	0 (0)	0 (0)	1 (0.8)	0 (0)	0 (0)
Dysuria	0 (0)	0 (0)	0 (0)	0 (0)	0 (0)	1 (0.8)	0 (0)	0 (0)
**Reproductive system and breast disorders**	0 (0)	2 (1.7)	0 (0)	2 (1.7)	0 (0)	1 (0.8)	2 (1.7)	1 (0.8)
Breast inflammation	0 (0)	0 (0)	0 (0)	0 (0)	0 (0)	0 (0)	1 (0.8)	0 (0)
Dysfunctional uterine bleeding	0 (0)	1 (0.8)	0 (0)	0 (0)	0 (0)	0 (0)	0 (0)	0 (0)
Dysmenorrhea	0 (0)	0 (0)	0 (0)	1 (0.8)	0 (0)	0 (0)	0 (0)	1 (0.8)
Erectile dysfunction	0 (0)	1 (0.8)	0 (0)	0 (0)	0 (0)	0 (0)	0 (0)	0 (0)
Genital ulceration	0 (0)	0 (0)	0 (0)	0 (0)	0 (0)	1 (0.8)	1 (0.8)	0 (0)
Uterine hemorrhage	0 (0)	0 (0)	0 (0)	1 (0.8)	0 (0)	0 (0)	0 (0)	0 (0)
**Respiratory, thoracic and mediastinal disorders**	9 (7.5)	5 (4.2)	6 (5.0)	7 (5.8)	7 (5.8)	9 (7.5)	3 (2.5)	10 (8.3)
Asthma	0 (0)	0 (0)	1 (0.8)	0 (0)	0 (0)	0 (0)	0 (0)	1 (0.8)
Cough	4 (3.3)	3 (2.5)	2 (1.7)	0 (0)	3 (2.5)	7 (5.8)	1 (0.8)	3 (2.5)
Epistaxis	0 (0)	0 (0)	0 (0)	1 (0.8)	0 (0)	0 (0)	0 (0)	0 (0)
Productive cough	0 (0)	0 (0)	1 (0.8)	0 (0)	0 (0)	0 (0)	0 (0)	0 (0)
Rhinitis allergic	0 (0)	1 (0.8)	0 (0)	2 (1.7)	1 (0.8)	0 (0)	0 (0)	2 (1.7)
Rhinorrhea	5 (4.2)	1 (0.8)	3 (2.5)	3 (2.5)	3 (2.5)	1 (0.8)	2 (1.7)	4 (3.3)
Throat irritation	0 (0)	0 (0)	0 (0)	0 (0)	0 (0)	1 (0.8)	0 (0)	0 (0)
Tonsillar inflammation	0 (0)	0 (0)	0 (0)	1 (0.8)	0 (0)	0 (0)	0 (0)	0 (0)
**Skin and subcutaneous tissue disorders**	3 (2.5)	2 (1.7)	5 (4.2)	4 (3.3)	3 (2.5)	3 (2.5)	3 (2.5)	5 (4.2)
Dermatitis	1 (0.8)	0 (0)	1 (0.8)	2 (1.7)	0 (0)	0 (0)	0 (0)	2 (1.7)
Dermatitis allergic	2 (1.7)	0 (0)	2 (1.7)	0 (0)	1 (0.8)	2 (1.7)	1 (0.8)	0 (0)
Dermatitis contact	0 (0)	0 (0)	0 (0)	0 (0)	0 (0)	0 (0)	1 (0.8)	0 (0)
Itching scar	0 (0)	1 (0.8)	0 (0)	0 (0)	0 (0)	0 (0)	0 (0)	0 (0)
Night sweats	0 (0)	0 (0)	0 (0)	0 (0)	0 (0)	0 (0)	1 (0.8)	0 (0)
Pruritus	0 (0)	1 (0.8)	2 (1.7)	0 (0)	0 (0)	1 (0.8)	0 (0)	3 (2.5)
Pruritus generalized	0 (0)	0 (0)	0 (0)	1 (0.8)	0 (0)	0 (0)	0 (0)	0 (0)
Skin irritation	0 (0)	0 (0)	0 (0)	0 (0)	1 (0.8)	0 (0)	0 (0)	0 (0)
Skin lesion	0 (0)	0 (0)	0 (0)	0 (0)	1 (0.8)	0 (0)	0 (0)	0 (0)

Urticaria	0 (0)	0 (0)	0 (0)	1 (0.8)	0 (0)	0 (0)	0 (0)	0 (0)

MedDRA version 20.0

Table S15: Severity of adverse events up to Day 28 post-vaccination by study arm, and vaccine manufacturer, safety population

**Severity**	**Bio-Manguinhos**		**Chumakov IPVE**		**Institut Pasteur Dakar**		**Sanofi Pasteur**	
n (%) with ≥ 1 AE	Frac. N=120	Standard N=120	Frac. N=119	Standard N=120	Frac. N=120	Standard N=120	Frac. N=120	Standard N=120
Mild	57 (47.5)	66 (55.0)	65 (54.6)	67 (55.8)	73 (60.8)	69 (57.5)	54 (45.0)	69 (57.5)
Moderate	4 (3.3)	7 (5.8)	8 (6.7)	11 (9.2)	9 (7.5)	6 (5.0)	10 (8.3)	9 (7.5)
Severe	0 (0)	0 (0)	0 (0)	0 (0)	0 (0)	0 (0)	0 (0)	0 (0)
Life Threatening	0 (0)	0 (0)	0 (0)	0 (0)	0 (0)	0 (0)	0 (0)	0 (0)

Table S16: Proportion of participants with at least one related event up to Day 28 post-vaccination, by study arm, and vaccine manufacturer, safety population

**Relatedness**	**Bio-Manguinhos**		**Chumakov IPVE**		**Institut Pasteur Dakar**		**Sanofi Pasteur**	
n (%) with ≥ 1 AE	Frac. N=120	Standard N=120	Frac. N=119	Standard N=120	Frac. N=120	Standard N=120	Frac. N=120	Standard N=120
Related	38 (31.7)	50 (41.7)	55 (46.2)	55 (45.8)	59 (49.2)	50 (41.7)	48 (40.0)	53 (44.2)

Table S17: Outcome of adverse events up to Day 28 post-vaccination, by study arm, and vaccine manufacturer, safety population

**Outcome**	**Bio-Manguinhos**		**Chumakov IPVE**		**Institut Pasteur Dakar**		**Sanofi Pasteur**	
n (%) with ≥ 1 AE	Frac. N=120	Standard N=120	Frac. N=119	Standard N=120	Frac. N=120	Standard N=120	Frac. N=120	Standard N=120
Recovered	58 (48.3)	69 (57.5)	69 (58.0)	72 (60.0)	76 (63.3)	72 (60.0)	60 (50.0)	70 (58.3)
Recovered with sequelae	0 (0)	0 (0)	0 (0)	0 (0)	0 (0)	0 (0)	0 (0)	0 (0)

Serious Adverse Events

Table S18: Summary of serious adverse events by study arm, and vaccine manufacturer, safety population

	Bio-Manguinhos		Chumakov IPVE		Institut Pasteur Dakar		Sanofi Pasteur	
	Frac. N=120	Standard N=120	Frac. N=119	Standard N=120	Frac. N=120	Standard N=120	Frac. N=120	Standard N=120
n (%) with ≥ 1 SAE	2 (1.7)	1 (0.8)	0 (0)	0 (0)	3 (2.5)	2 (1.7)	0 (0)	2 (1.7)

Table S19: Serious adverse events by MedDRA (v20.0) coding by study arm and vaccine manufacturer, safety population

System Organ Class and Preferred Term	Bio-Manguinhos		Chumakov IPVE		Institut Pasteur Dakar		Sanofi Pasteur	
n (%) with ≥ 1 SAE	Frac. N=120	Standard N=120	Frac. N=119	Standard N=120	Frac. N=120	Standard N=120	Frac. N=120	Standard N=120
**General disorders and administration site conditions**	0 (0)	0 (0)	0 (0)	0 (0)	0 (0)	1 (0.8)	0 (0)	0 (0)
Sudden death	0 (0)	0 (0)	0 (0)	0 (0)	0 (0)	1 (0.8)	0 (0)	0 (0)
**Infections and infestations**	1 (0.8)	0 (0)	0 (0)	0 (0)	0 (0)	1 (0.8)	0 (0)	1 (0.8)
Malaria	1 (0.8)	0 (0)	0 (0)	0 (0)	0 (0)	1 (0.8)	0 (0)	0 (0)
Wound sepsis	0 (0)	0 (0)	0 (0)	0 (0)	0 (0)	0 (0)	0 (0)	1 (0.8)
**Injury, poisoning, and procedural complaints**	0 (0)	0 (0)	0 (0)	0 (0)	1 (0.8)	0 (0)	0 (0)	0 (0)
Road traffic accident	0 (0)	0 (0)	0 (0)	0 (0)	1 (0.8)	0 (0)	0 (0)	0 (0)
**Pregnancy, puerperium and perinatal conditions**	0 (0)	0 (0)	0 (0)	0 (0)	1 (0.8)	0 (0)	0 (0)	0 (0)
Spontaneous incomplete abortion	0 (0)	0 (0)	0 (0)	0 (0)	1 (0.8)	0 (0)	0 (0)	0 (0)
**Respiratory, thoracic and mediastinal disorders**	0 (0)	1 (0.8)	0 (0)	0 (0)	0 (0)	0 (0)	0 (0)	0 (0)
Lung neoplasm malignant	0 (0)	1 (0.8)	0 (0)	0 (0)	0 (0)	0 (0)	0 (0)	0 (0)
**Surgical and medical procedures**	1 (0.8)	0 (0)	0 (0)	0 (0)	1 (0.8)	0 (0)	0 (0)	1 (0.8)
Caesarean section	1 (0.8)	0 (0)	0 (0)	0 (0)	1 (0.8)	0 (0)	0 (0)	1 (0.8)

MedDRA version 20.0

Table S20: Outcome of serious adverse events, by study arm, and vaccine manufacturer, safety population

Outcome	Bio-Manguinhos		Chumakov IPVE		Institut Pasteur Dakar		Sanofi Pasteur	
n (%) with ≥ 1 SAE	Frac. N=120	Standard N=120	Frac. N=119	Standard N=120	Frac. N=120	Standard N=120	Frac. N=120	Standard N=120
Recovered	2 (1.7)	0 (0)	0 (0)	0 (0)	2 (1.7)	1 (0.8)	0 (0)	1 (0.8)
Recovered with sequelae	0 (0)	0 (0)	0 (0)	0 (0)	0 (0)	0 (0)	0 (0)	1 (0.8)
Fatal or died	0 (0)	1 (0.8)	0 (0)	0 (0)	1 (0.8)	1 (0.8)	0 (0)	0 (0)
